# ZNHIT3 Regulates Translation to Ensure Cell Lineage Differentiation in Mouse Preimplantation Development

**DOI:** 10.1002/advs.202413599

**Published:** 2025-04-03

**Authors:** Guanghui Yang, Qiliang Xin, Jurrien Dean

**Affiliations:** ^1^ Laboratory of Cellular and Developmental Biology NIDDK National Institutes of Health Bethesda MD 20892 USA; ^2^ Present address: Howard Hughes Medical Institute Department of Biology The Johns Hopkins University 3400 North Charles Street Baltimore MD 21218 USA

**Keywords:** box C/D snoRNA, embryo development, first cell fate commitment, ribosomal RNA, SMARM‐seq, snoRNP complex, translation, ZNHIT3

## Abstract

Upon fertilization, the mouse zygotic genome is activated and maternal RNAs as well as proteins are degraded. Early developmental programs are built on proteins encoded by zygotic mouse genes that are needed to guide early cell fate commitment. The box C/D snoRNA ribonucleoprotein (snoRNP) complex is required for rRNA biogenesis, ribosome assembly and pre‐mRNA splicing essential for protein translation. Zinc finger, HIT type 3 (encoded by *Znhit3*) is previously identified as a component in the assembly of the box C/D snoRNP complex. Using gene‐edited mice, it identifies *Znhit3* as an early embryonic gene whose ablation reduces protein translation and prevents mouse embryos development beyond the morula stage. The absence of ZNHIT3 leads to decreased snoRNA and rRNA abundance which causes defects of ribosomes and mRNA splicing. Microinjection of *Znhit3* cRNA partially rescues the phenotype and confirms that ZNHIT3 is required for mRNA translation during preimplantation development.

## Graphical abstract

Box C/D snoRNP complex regulates protein translation. This study identifies ZNHIT3 as a key component of the complex and ensures normal protein expression, including transcription factors orchestrating cell fate commitment, during early mouse embryogenesis. *Znhit3* ^−^ *^/^* ^−^ embryos show abnormality caused by translation machinery defeats and arrest at the morula stage which could be rescued by microinjection of *Znhit3* cRNA.

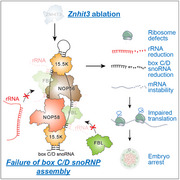

## 1 Introduction

Proper spatial and temporal protein expression is key to the function of multicellular organisms in early development.^[^ ^1^ ^]^ Over or under production of transcription factors is sufficient to change lineage, thus protein expression is crucial in determining mammalian cell fate.^[^ ^2^ ^]^ Upon fertilization, two terminally differentiated mammalian gametes fuse to form a totipotent zygote that divides and differentiates. As early as 2‐cells, there are differences between blastomeres that subsequently generate cell lineages.^[^ ^3^ ^]^ The maternal‐zygotic transition occurs within 24 h of fertilization in mice and there is concomitant degradation of maternal transcripts^[^ ^4^ ^]^ and zygotic gene activation (ZGA).^[^ ^5^ ^]^ Heretofore, considerable emphasis has been placed on gene expression during this transition with a minor burst at the late 1‐cell stage and major, more robust gene activation at the 2‐cell stage in mice.^[^ ^6^ ^]^ However, translation of coding transcripts into proteins has increasingly been documented to play a role in this transition from maternal to zygotic control.

Inhibition of protein synthesis based on maternal transcripts after fertilization but before zygotic gene expression arrests embryos at the 1‐cell stage which documents the need for *de novo* proteins in preimplantation development.^[^ ^7^ ^]^ Recently a germ‐cell specific initiation factor, eIF4E1b has been documented to be required to progress beyond the 2‐cell stage of mouse development.^[^ ^8^ ^]^ The mouse embryos utilize eIF4E1b to translate a selective group of maternal transcripts whose products are needed for zygotic genome activation. After the major wave of ZGA at embryonic day 2 (E2.0), the embryonic genome controls preimplantation development. As early as E3.0, two distinguishable lineages are present in the mouse embryos.^[^ ^9^ ^]^ The short time window again requires rapid accumulation of corresponding zygotic proteins.^[^ ^10^ ^]^ However, the underlying mechanisms remain unclear.

Ribosomes are factories for protein translation and form ribonucleoprotein complexes (RNP) containing mRNA, ribosome RNA (rRNA), non‐coding RNA (ncRNA) and proteins.^[^ ^11^ ^]^ After the major ZGA at E2.0, translational efficiency increases during preimplantation development and is required for cell fate commitment.^[^ ^10^, ^12^ ^]^ Small nucleolar RNAs (snoRNAs) form a subset of abundantly expressed ncRNAs and box C/D snoRNAs (SNORDs) represent a large subtype of snoRNAs. Like other snoRNAs, SNORDs function in box C/D snoRNA ribonucleoprotein complexes (box C/D snoRNP or SNORNP) which incorporate SNORD and associated protein components. SNORNPs participate in RNA biogenesis that includes rRNA modification, pre‐mRNA splicing and RNA stability.^[^ ^13^ ^]^ Box C/D snoRNP assembly is sequential in which individual proteins have been identified.^[^ ^14^ ^]^ A heterodimer containing fibrillarin (FBL), NOP56, NOP58 and SNU13 proteins bind a snoRNA to form the core functional box C/D snoRNP complex. Its assembly requires assistance from several proteins, including ZNHIT3, NUFIP1 and PIH1D1. Deletion of fibrillarin protein caused failure of early mouse development.^[^ ^15^ ^]^

However, there is limited data on why *Fibrillarin* null mice arrest during early embryogenesis nor is there insight into factors that regulate formation of the box C/D snoRNP complex. The striking phenotype in the absence of fibrillarin prompted us to explore the regulatory mechanisms of box C/D snoRNP complex formation during early mouse development. In the current work, we identify *Znhit3* as an early embryonic gene that affects protein translation by controlling snoRNA and rRNA abundance and pre‐mRNA splicing. It is essential for mouse preimplantation development and homozygous null mutant embryos (*Znhit3^Null^*) arrest at the morula stage.

## 2 Results

### 2.1 *Znhit3* is an Early Zygotic Gene Conserved Among Mammals

**Figure 1:**
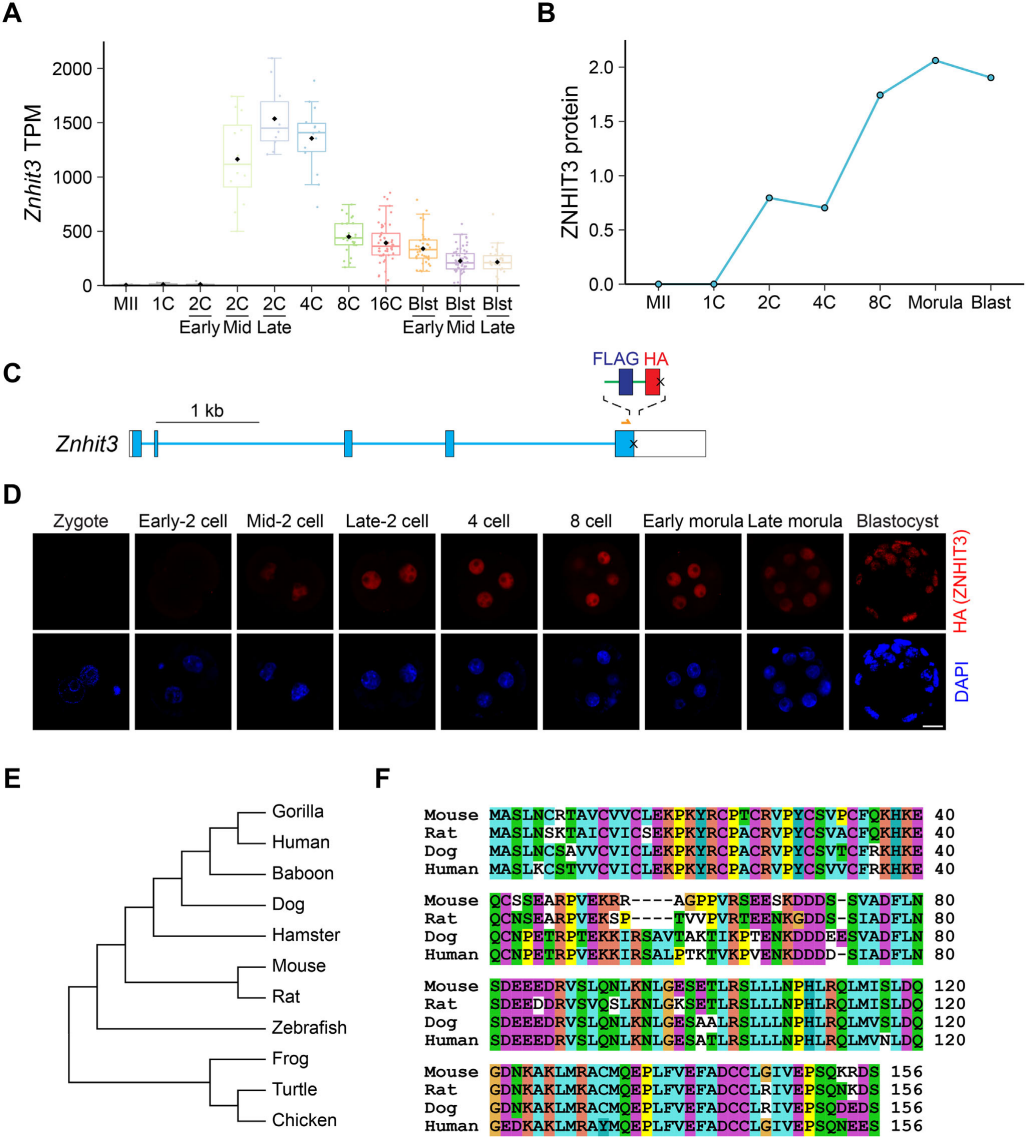
*Znhit3* is an early zygotic gene that expresses in mammals. A) Abundance (TPM, transcripts per million mapped reads) of *Znhit3* during early mouse embryo development.^[^ ^16^ ^]^ B) Normalized protein level of ZNHIT3 from published proteomics results during early mouse embryo development.^[^ ^7a^ ^]^ C) Schematic of the *Znhit3* gene locus in the *Znhit3^KI^* line with FLAG and HA tags at the C terminus. Yellow arrow indicates position of the guide RNA and “x” for stop codon. D) Immunofluorescence of preimplantation embryos derived from *Znhit3^KI^* female mice. Anti‐HA antibody is used to visualize the ZNHIT3 fusion protein whose signal is quantified in Figure S1E (Supporting Information); DAPI for nuclear DNA. Scale bar, 20 µm. E) Phylogenetic analysis using ZNHIT3 protein sequences from selected vertebrates. The evolutionary tree was generated by MEGA.^[^ ^21^ ^]^ F) Alignment of mouse ZNHIT3 protein sequence with that of multiple mammals using Clustal X.^[^ ^22^ ^]^

To identify potential regulators of box C/D snoRNP during early embryo development, we examined expression of genes involved in box C/D snoRNP complex assembly using published single cell RNA‐seq (scRNA‐seq) data.^[^ ^16^ ^]^ Although *Fbl* deletion caused failure of early embryogenesis,^[^ ^15^ ^]^ *Fbl* expression in mouse blastomeres does not show obvious differences among developmental stages (Figure S1A, Supporting Information). This suggests that *Fbl* may not regulate formation of the box C/D snoRNP complex, although incorporated into its functional core. There was also no differential expression of other genes involved in box C/D snoRNP assembly save for *Znhit3* (Figure S1A, Supporting Information). ZNHIT3 was previously identified as a component of box C/D snoRNP complex from mass spectrometry profiling.^[^ ^17^ ^]^ However, it is unknown how ZNHIT3 regulates box C/D snoRNP functions. *Znhit3* mRNA is barely detected in mature mouse oocytes but increases dramatically in blastomeres at the mid 2‐cell stage (**Figure** **1**A) when expression of the first embryonic genes are detected.^[^ ^6^ ^]^ Human *ZNHIT3* shows similar expression pattern whose level significantly increases at the 8‐cell stage, at which time the human zygotic genome activation occurs (Figure S1B, Supporting Information), suggesting similar functions in embryogenesis.^[^ ^18^ ^]^ *Znhit3* mRNA remains highly abundance in blastomeres throughout preimplantation development. The dramatic increase makes *Znhit3* an attractive candidate for regulating box C/D snoRNP complex assembly (Figure 1A). ZNHIT3 protein levels had a similar profile during mouse early embryo development as confirmed by quantitative proteomics analysis (Figure 1B).^[^ ^7a^ ^]^

We established a knock‐in (*Znhit3^KI^*) mouse line in which FLAG (DYKDDDDK) and HA (HemAgglutinin) tags were inserted at the carboxyl terminus of the protein (Figure 1C; Figure S1C,D, Supporting Information) to confirm that ZNHIT3 was present in the mid 2‐cell embryo and persisted throughout preimplantation development (Figure 1D; Figure S1E, Supporting Information). ZNHIT3 is an evolutionary conserved protein present in yeast and mammals (Figure 1E).^[^ ^19^ ^]^ However, even among mammals within a short evolutionary distance, the ZNHIT3 protein sequences have multiple non‐conserved amino acids (Figure 1F). Comparison of ZNHIT3 protein sequence against the entire mouse protein sequence database^[^ ^20^ ^]^ identified no proteins with high sequence similarity. These results suggest that *Znhit3* is a single copy zygotic gene that may play a highly specialized function during early mammalian development.

### 2.2 *Znhit3* Null Embryos Fail to Progress Beyond the Morula Stage of Embryogenesis

**Figure 2:**
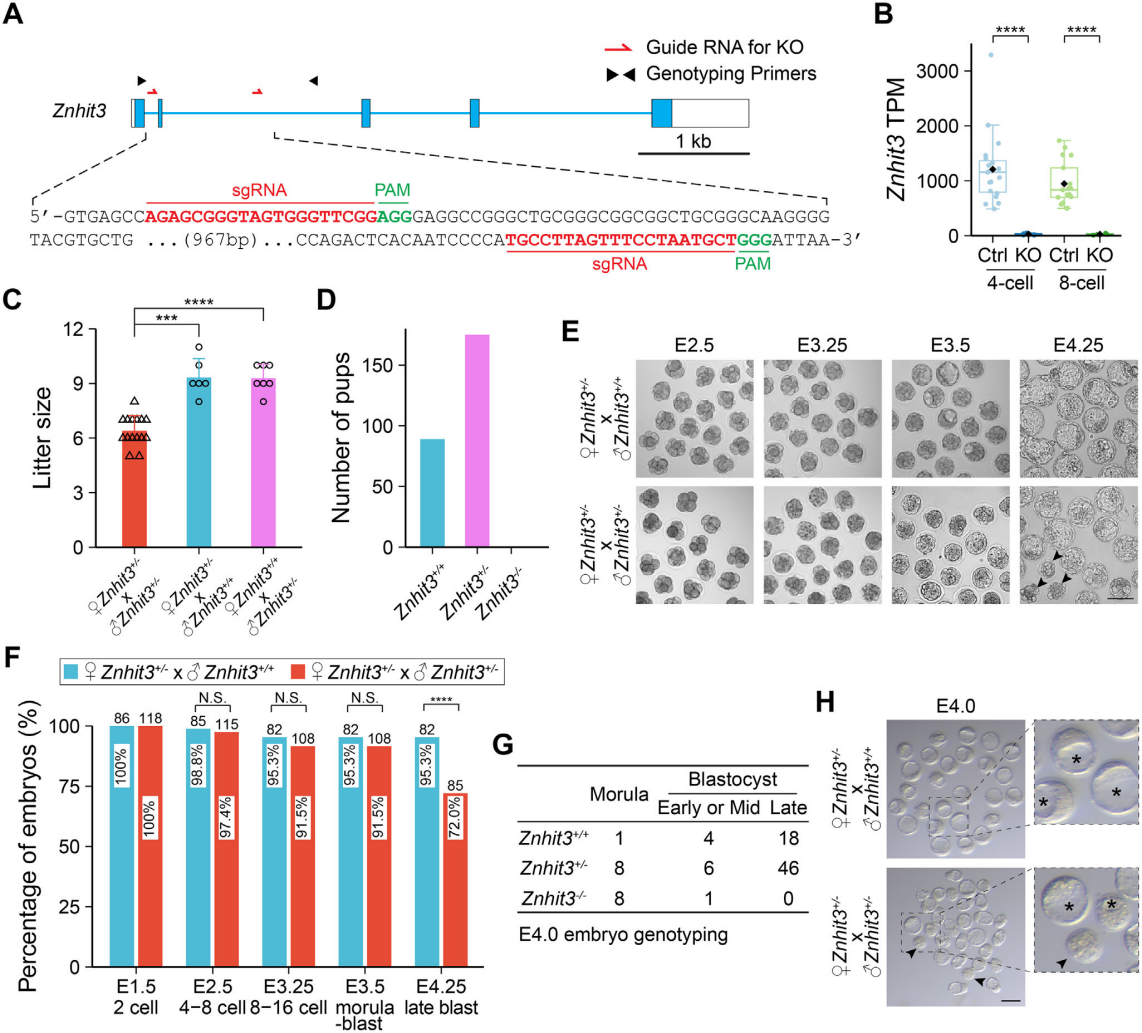
*Znhit3* homozygous null embryos arrest at morula stage. A) Schematic of *Znhit3* gene and sequences of sgRNAs for generation of *Znhit3^KO^* mouse line. sgRNA and genotyping primer positions are indicated. B) Abundance of *Znhit3* transcripts (TPM) in *Znhit3^KO^* and control embryos as determined by seRNA‐seq. C) Litter sizes of different matings. D) Genotypes of accumulated pups after intercross of *Znhit3^+/−^* mice. E) Representative images of in vitro cultured embryos after intercross of *Znhit3^+/−^* mice. Arrow heads indicate degenerated embryos. Scale bar, 100 µm. F) Quantification of embryos in E. Ratio of embryos at different stages is plotted. Total number of embryos are on top of each bar. G) Genotypes of in vitro cultured embryos at E4.0 after intercross of *Znhit3^+/−^* mice. H) Images of embryos flushed from uterus after in vivo mating of *Znhit3^+/−^* mice. Arrow heads indicate degenerated *Znhit3^−/−^* embryos. Asterisks labels blastocoels. Scale bar, 100 µm.

To confirm *Znhit3* functions during early embryo development, we established mouse lines with CRISPR/Cas9 in which one allele of *Znhit3* was ablated (**Figure** **2**A). The establishment of a *Znhit3* null allele in heterogenous (*Znhit3^+/−^*) founder mice was confirmed by DNA sequence (Figure S1F, Supporting Information). No *Znhit3^−/−^* homozygous founders were obtained. After multiple backcrosses to ensure a congenic strain, *Znhit3^+/−^* mice were intercrossed. Based on single embryo RNA‐seq (seRNA‐seq) (Figure S2A, Supporting Information), no *Znhit3* transcripts could be detected in *Znhit3^−/−^* embryos (Figure 2B). The intercross mating pairs had statistically significant smaller litter sizes compared to those mated with wildtype (WT) controls (Figure 2C). We confirmed the reduced litter size was due to absent *Znhit3* homozygous null (*Znhit3^−/−^*) pups (Figure 2D), suggesting ZNHIT3 is essential for successful embryogenesis. To determine the onset of defects in *Znhit3^−/−^* embryos, zygotes were flushed after intercross of *Znhit3^+/−^* mice and cultured in vitro for 4 days. Zygotes from *Znhit3^+/−^* females after mating with WT males were used as controls (Figure 2E). Compared to controls, degeneration was observed in embryos from *Znhit3^+/−^* intercross beginning at the morula stage. While all the control embryos grew into blastocysts ready to hatch from the zona pellucida at E4.25, ≈25% of the embryos from *Znhit3^+/−^* intercross were already degenerated or retained morphology of morulae (Figure 2E,F). We thus suspected the onset of embryo defects happened before E4.25 and genotyped single embryos at E4.0 after *Znhit3^+/−^* intercross and in vitro culture. Eight of the 9 *Znhit3^−/−^* embryos detected remained as morulae while *Znhit3^+/−^* or WT (*Znhit3^+/+^*) embryos progressed to blastocysts (Figure 2G). This developmental defect of *Znhit3^−/−^* embryos also occurs in vivo as determined by flushing embryos from oviduct at E4.0 after mating (Figure 2H; Figure S1G, Supporting Information). Taking together, our results document ZNHIT3 is essential for mouse embryos to develop beyond the morula stage.

### 2.3 *Znhit3* Ablation Causes Global Decrease in Abundance of Transcripts in Morulae

**Figure 3:**
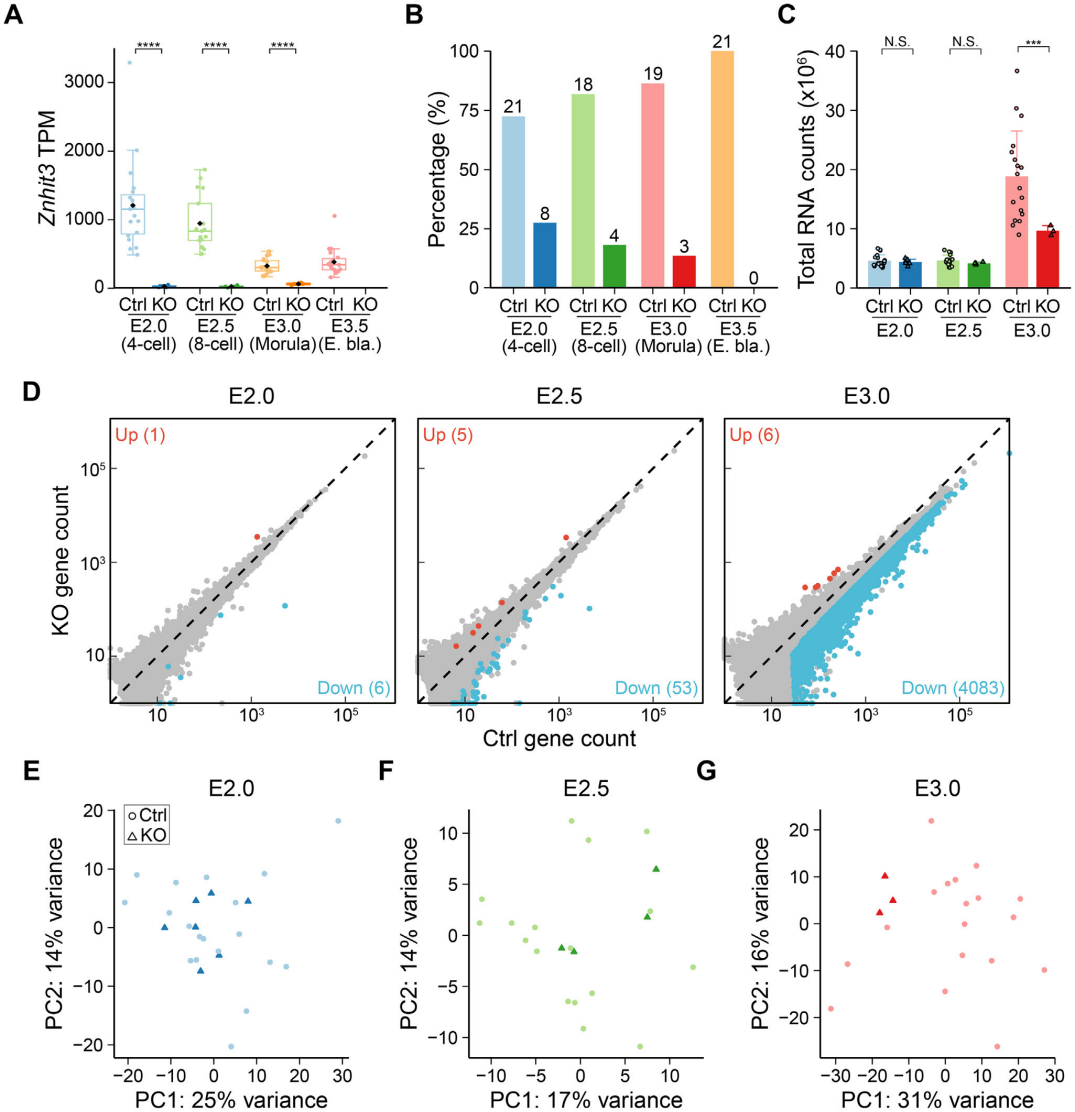
Analyses of comparative seRNA‐seq data between *Znhit3^−/−^* and control embryos after mating and in vitro culture. A) TPM of *Znhit3* gene for each embryo collected at different developmental stages. The TPM values are used to confirm the genotype of each embryo as homozygous null (*Znhit3^−/−^*) or control (Ctrl). No *Znhit3^−/−^* embryos progressed to blastocysts. B) Percentage of *Znhit3^−/−^* or control embryos at different developmental stages after culture in vitro. C) Abundance of total RNA counts in *Znhit3^−/−^* and control embryos at different developmental stages. ERCC spike‐ins are used for normalization. Each dot represents one embryo, and standard errors are shown. D) Scatter plots showing expression of genes in *Znhit3^−/−^* and control embryos at different developmental stages. Differential expressed genes are labeled as red or blue dots with their total number indicated. Note, many more genes are down regulated in *Znhit3^−/−^* embryos, especially at E3.0 when control embryos have become morulae. (E‐G) Principal component analysis (PCA) plot of seRNA‐seq results of *Znhit3^−/−^* or controls at different developmental stages. Note the *Znhit3^−/−^* embryos are difficult to distinguish from controls up to E2.5 while they separate quite well at E3.0.

To investigate the molecular basis for the morula arrest after ablation of *Znhit3*, we performed seRNA‐seq after intercross of *Znhit3^+/−^* mice by adapting the single cell G&T‐seq protocol^[^ ^23^ ^]^ to individual embryos (Figure S2A and Table S1, Supporting Information). Most (≈83%) annotated mRNAs were identified in all samples indicating successful poly(A) capture and sequencing (Figure S2B, Supporting Information). Using the abundance of *Znhit3* transcripts in single embryos (**Figure** **3**A), we calculated the ratio of *Znhit3^−/−^* embryos at different stages (Figure 3B). Although the percentage of *Znhit3^−/−^* embryos followed Mendelian ratios at late 4‐cell stage, we began to observe reduced number of *Znhit3^−/−^* embryos as early as the 8‐cell stage and no *Znhit3^−/−^* blastocysts were retrieved from intercross of *Znhit3^+/−^* mice. These results further confirmed the morula arrest phenotype that we observed (Figure 2F‐H). Although the morulae did not show obvious degeneration, *Znhit3^−/−^* embryos had significantly decreased abundance of many transcripts with very few up regulated genes (Figure 3C,D; Figure S2C, Supporting Information). These results suggest extensive degeneration of transcripts in *Znhit3^−/−^* morulae, agreeing with the observed morula arrest. Similarly, many more transcripts were down regulated compared to those that were up regulated in *Znhit3^−/−^* 4‐ and 8‐cell embryos, although not as dramatically as at the morula stage (Figure 3D). Agreeing with these observations, early (4‐ or 8‐cell) *Znhit3^−/−^* embryos are difficult to distinguish from control embryos while *Znhit3^−/−^* morulae are morphologically distinct from controls (Figures 2E‐H and 3E‐G). Taken together, these results are consistent with RNA degradation that becomes more severe as the *Znhit3^−/−^* embryos progress into the morula stage and leads to embryonic arrest.

**Figure 4:**
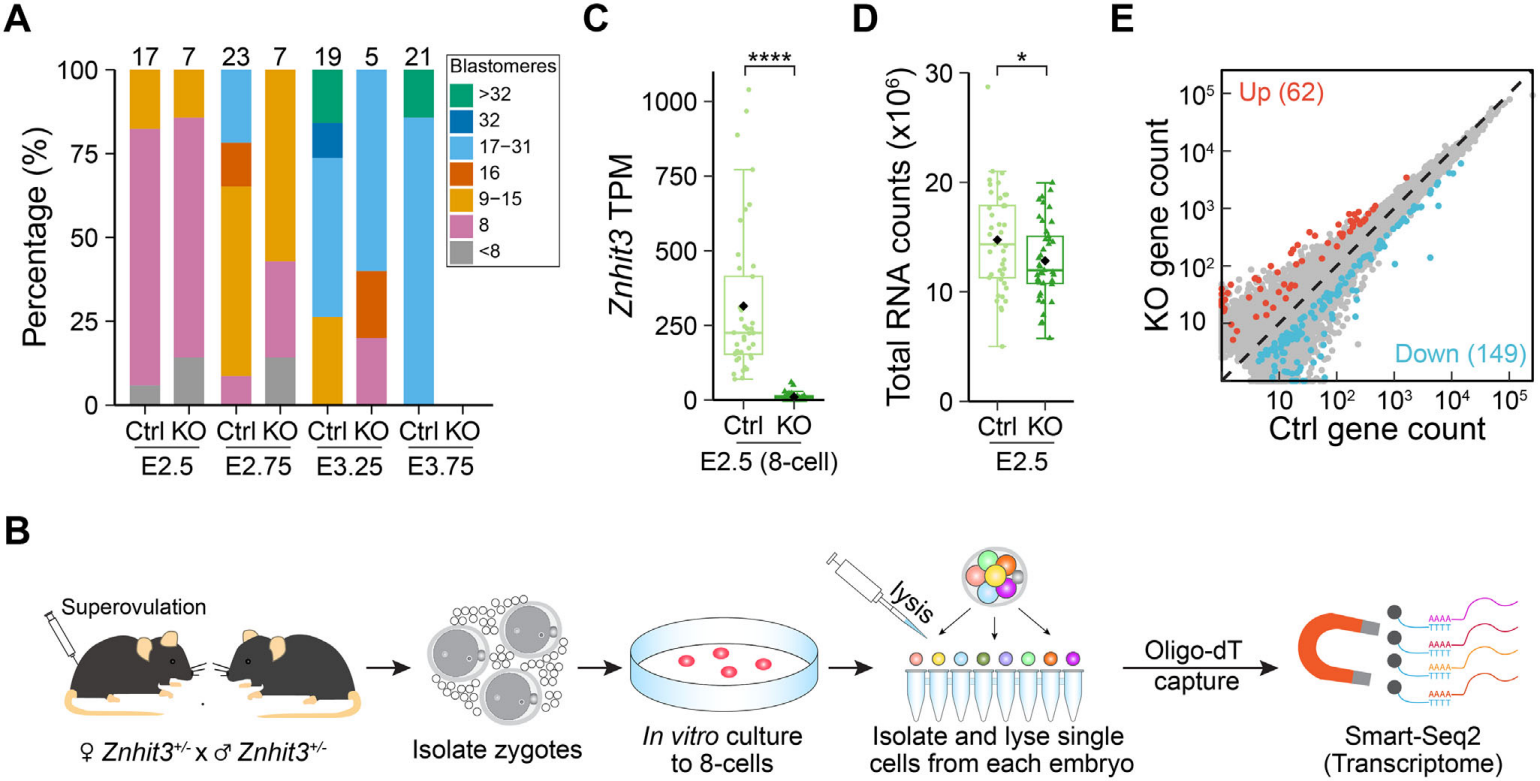
Analysis of blastomeres from *Znhit3^−/−^* or control embryos. A) Statistics of blastomere numbers in each embryo at different development stages. Percentage of embryos with different number of blastomeres are shown in the bar plot with total number of embryos examined on top. The experiment was repeated thrice with one replicate displayed. B) Scheme of the scRNA‐seq. Single blastomeres isolated from 8‐cell embryos after intercross of *Znhit3^+/−^* breeders are sequenced following the Smart‐Seq2 protocol. C) Expression of *Znhit3* in each blastomere. ERCC normalized TPM values of *Znhit3* is used to distinguish homozygous null (*Znhit3^−/−^*) blastomeres from control (Ctrl). D) Total amount of RNA in *Znhit3^−/−^* or Ctrl 8‐cell blastomeres. ERCC normalized total RNA counts are shown. E) Scatter plot showing expression of genes in *Znhit3^−/−^* and control 8‐cell blastomeres. Differential expressed genes are labeled as red or blue dots with their total number indicated.

To determine the mechanism(s) that caused global down regulation of transcripts in *Znhit3^−/−^* morulae, the knock‐in mouse line (*Znhit3^KI^*) was crossed with *Znhit3^+/−^* mice to generate a new *Znhit3^KI/−^* line. *Znhit3^KI/−^* mice were then intercrossed, so that we could retrieve *Znhit3^−/−^* embryos as well as control embryos (*Znhit3^KI/KI^* or *Znhit3^KI/−^*) after in vitro culture of zygotes. Using anti‐HA immunofluorescence, we could distinguish control from *Znhit3* null embryos. We calculated the total number of blastomeres in each embryo and found that starting from E2.5 when most control embryos had become 8‐cell embryos, the homozygous *Znhit3* null embryos contained fewer blastomeres in each embryo. The reduction in blastomeres became more pronounced at E2.75 and E3.25 at which time control embryos had progressed to the morulae (**Figure** **4**A). This observation was confirmed by examination of in vivo flushed embryos at E3.5 after intercrossing of *Znhit3^KI/−^* mice (Figure S1H, Supporting Information). These results firmly documented arrest of blastomere growth and viability which ultimately led to developmental arrest at the morula stage of *Znhit3^−/−^* embryos.

To ascertain if the decrease in the abundance of transcripts reflected reduced number of blastomeres in *Znhit3^−/−^* embryos or if there were additional defects in the remaining blastomeres, we cultured zygotes in vitro and collected single blastomeres from embryos at E2.5. At this stage, most *Znhit3^−/−^* and all control embryos could develop into 8‐cell embryos. The single blastomeres were then used for scRNA‐seq (Figure 4B; Table S2, Supporting Information). The capture of transcripts with poly(A) tails from single blastomeres was efficient (Figure S3A, Supporting Information) and confirmed success of the scRNA‐seq technology. We confirmed genotypes of blastomeres based on abundance of *Znhit3* transcripts (Figure 4C) and found that the amount of poly(A) RNA from *Znhit3^−/−^* 8‐cell blastomeres was decreased (Figure 4D), although not as much as in *Znhit3^−/−^* morulae (Figure 3D). More genes were significantly down regulated in *Znhit3^−/−^* 8‐cell blastomeres than in blastomeres from control 8‐cell embryos (Figure 4E). As more samples (single blastomeres) were applied for scRNA‐seq and thus improved the statistical power, we were also able to identify more differential expressed genes in *Znhit3^−/−^* 8‐cell blastomeres than in 8‐cell embryos from seRNA‐seq (Figure 3D). These results suggest the ablation of *Znhit3* causes global defects in gene expression as early as the 8‐cell stage and is accelerated during preimplantation development. In agreement with the obvious yet still minor defects observed at 8‐cell stage, 8‐cell blastomeres from *Znhit3^−/−^* and control embryos could not be distinguished by principal component analysis (PCA) using scRNA‐seq data (Figure S3B, Supporting Information), and embryo degeneration was not obvious at this stage (Figure 2E,F). However, deficits increase over time in the *Znhit3* null embryos, and they ultimately arrest at the morula stage. Embryos degenerate after prolonged in vitro culture.

### 2.4 ZNHIT3 Deletion Reduces Transcription Factors Required for the First Cell Fate Commitment

**Figure 5:**
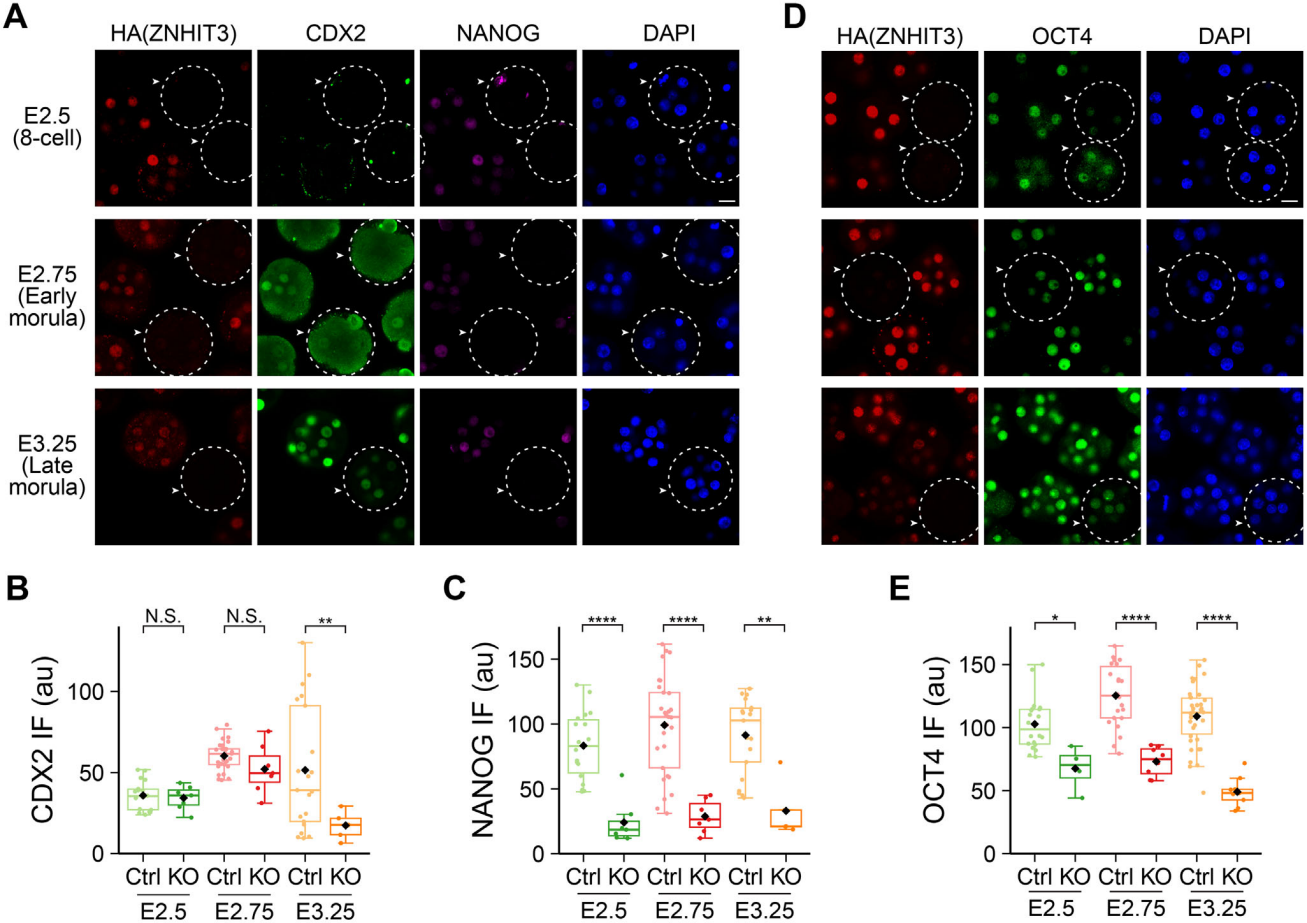
Depletion of ZNHIT3 leads to reduced expression of pluripotency factors in early embryos. A) CDX2 and NANOG protein levels in *Znhit3^−/−^* (arrowheads, dashed circles) and control (Ctrl) embryos at different developmental stages. B,C) Quantification of the nuclear fluorescent signals in A. D) OCT4 (encoded by *Pou5f1)* protein levels in *Znhit3^−/−^* (arrowheads, dashed circles) and control (Ctrl) embryos at different developmental stages. The nuclear fluorescent signals are quantified in E. Scale bar, 20 µm.

The phenotype of homozygous *Znhit3* ablation begins in the 8‐cell embryo and is exacerbated as embryos become morulae, at which time blastomeres begin to differentiate, and causes developmental arrest. Thus, we hypothesized that the arrest in blastomere and embryonic growth would be attributable to failure of embryo to differentiate. The first two distinguishable cell types in mouse embryos are the trophoblasts and the inner cell mass. This differentiation is controlled by two groups of antagonistic transcription factors: the pluripotency factors NANOG and OCT4 (encoded by *Pou5f1)* which drives formation of the inner cell mass and CDX2 which promotes trophoblast cell fate.^[^ ^9^ ^]^ We intercrossed *Znhit3^KI/−^* mice and examined expression of CDX2, NANOG and OCT4 in *Znhit3^−/−^* and control embryos at different developmental stages using immunofluorescence. CDX2 protein was barely detected until E2.75 when control embryos became early morulae. At this time point we could already detect reduced CDX2 abundance in homozygous *Znhit3* null embryos. CDX2 increased at E3.25 when control embryos had developed into the late‐morula stage. At this time point, CDX2 was significantly lower in *Znhit3^−/−^* embryos (**Figure** **5**A,B; Data S1, Supporting Information). NANOG and OCT4, two pluripotent factors controlling the first cell fate divergence in mouse embryos, could already be detected at E2.5 when control embryos were still 8‐cells (Figure 5A,D). We also detected significant decrease of NANOG and OCT4 in *Znhit3^−/−^* embryos at E2.5, E2.75 and E3.25 (Figure 5A, 5C‐E) similar to CDX2 (Figure 5A,B; Data S1, Supporting Information). These results suggest blastomere and embryo developmental arrest of *Znhit3^−/−^* embryos are not due to imbalance of the antagonistic program between CDX2 and pluripotency factors. Instead, a concomitant decrease of both CDX2 and the pluripotency factors could be the cause of the observed arrest.

### 2.5 In the Absence of ZNHIT3, Protein Synthesis is Reduced

**Figure 6:**
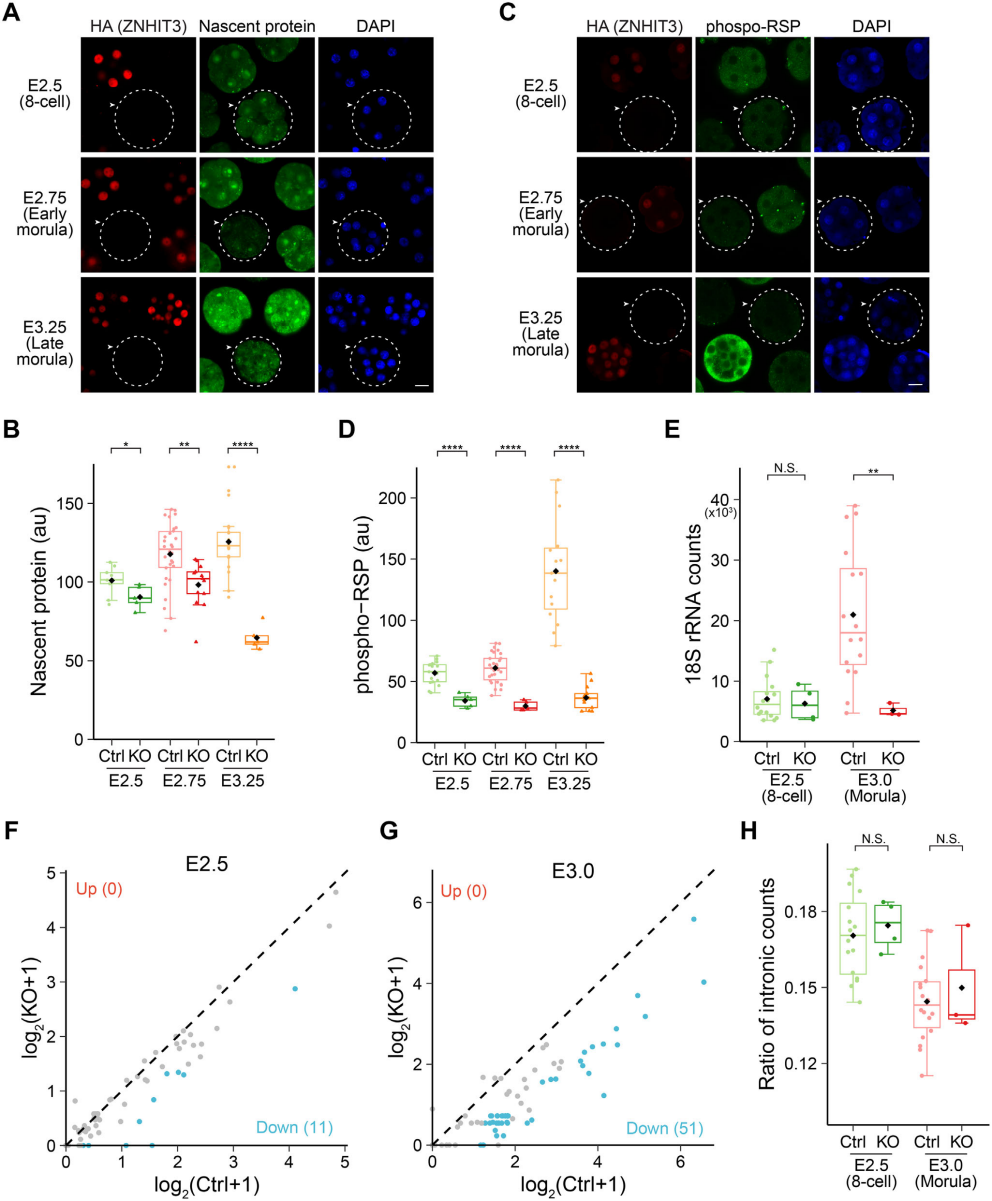
Ablation of *Znhit3* causes reduced protein synthesis in early embryos. A) Images of nascent proteins in *Znhit3^−/−^* (arrowheads, dashed circles) and control (Ctrl) embryos at different developmental stages. Scale bar, 20 µm. B) Quantification of the fluorescent signals in A. C) Images of phosphorylated S6 ribosomal protein (Ser235/236) in *Znhit3^−/−^* (arrowheads, dashed circles) and control (Ctrl) embryos at different developmental stages. Scale bar, 20 µm. D) Quantification of the fluorescent signals in C. E) Abundance of 18S rRNA in *Znhit3^−/−^* and control embryos at different developmental stages. F,G). Scatter plot showing abundance of box C/D snoRNA in *Znhit3^−/−^* and control embryos at different developmental stages. The box C/D snoRNAs that are significantly down regulated in the *Znhit3^−/−^* samples are labeled in blue with their total number specified. No box C/D snoRNAs are significantly up‐regulated in the *Znhit3^−/−^* samples. H) Ratio of intronic counts in seRNA‐seq data from *Znhit3^−/−^* and control embryos at different developmental stages.

CDX2 and pluripotency factors are negatively correlated in blastomeres during the first cell fate decision in early mouse embryos,^[^ ^24^ ^]^ but consistent decrease of these proteins was detected in *Znhit3^−/−^* 8‐cell and morula embryos. Surprisingly, we also found that although the *Znhit3^−/−^* embryos already showed significantly decreased abundance of NANOG and OCT4 proteins at the 8‐cell stage, transcripts of *Cdx2*, *Nanog* and *Oct4* did not have significant differences between *Znhit3^−/−^* and control embryos at this developmental stage as determined by seRNA‐seq (Figure S4A‐C, Supporting Information). This observation was confirmed by scRNA‐seq using 8‐cell blastomeres from *Znhit3^−/−^* and control embryos (Figure S4D‐F, Supporting Information). These observations led us to investigate whether there was defect in the protein synthesis in *Znhit3* null embryos. Indeed, *Znhit3^−/−^* embryos had reduced protein translation as early as E2.5 when control embryos were still 8‐cells. This defect became more pronounced at E2.75 and E3.25 at which time control embryos became morulae (**Figure** **6**A,B; Figure S4G,H and Data S1, Supporting Information). Thus, it appears that the reduced protein synthesis is the underlying mechanism for arrested blastomere growth and viability. We also examined phosphorylation of S6 ribosomal protein which labels active ribosomes.^[^ ^25^ ^]^ As expected, in these developmental stages, S6 ribosomal protein had decreased phosphorylation in homozygous *Znhit3^−/−^* embryos (Figure 6C,D; Data S1, Supporting Information) suggesting abnormal ribosome function. Taken together, these results support the hypothesis that *Znhit3* ablation impairs normal ribosome function which decreases protein synthesis in the homozygous null embryos and caused developmental arrest.

ZNHIT3 was previously reported to interact with many core component proteins in the box C/D snoRNP complex, using immunoprecipitation followed by mass spectrometry (IP/MS) in U2OS cells.^[^ ^17^ ^]^ We performed similar experiments by expressing FLAG tagged ZNHIT3 protein in mouse embryonic stem cells E14, followed by anti‐FLAG immunoprecipitation and mass spectrometry (IP/MS). NOP58, NUFIP1 and FBL as well as other core components of the box C/D snoRNP complex were identified in the IP/MS experiment, strongly support ZNHIT3 to be another key component of box C/D snoRNP complex in embryonic cells. (Table S3, Supporting Information). We further explored the subcellular localization of ZNHIT3 and NUFIP1 and confirmed they share very similar localization, further validating ZNHIT3 to be a key component of the box C/D snoRNP complex (Figure S5B, Supporting Information).

Box C/D snoRNP complex mediates 2′‐O‐methylation of rRNA that is essential for ribosome assembly and function.^[^ ^26^ ^]^ As a key regulator of box C/D snoRNP assembly, we investigated whether *Znhit3* ablation could affect 2′‐O‐methylation of rRNA. We adapted small RNA smart‐seq into the currently available RiboMeth‐seq method^[^ ^27^ ^]^ and established the small RNA smart‐seq facilitated RiboMeth‐seq (SMARM‐seq) to profile 2′‐O‐methylation of rRNA from groups of embryos (Figure S6A and Table S4, Supporting Information). With the newly established SMARM‐seq, we could determine the frequency of a rRNA base captured at the 5′ end of a sequencing read. This frequency was used to calculate the RiboMeth score which represents the level of 2′‐O‐methylation (Figure S6B, Supporting Information). We successfully detected many conserved rRNA bases (Figure S6C,D, Supporting Information) that were reported to be 2′‐O‐methylated in different species.^[^ ^27^, ^28^ ^]^ We also identified other rRNA sites that could be potential mouse specific 2′‐O‐methylated sites (Figure S6E, Supporting Information) while some rRNA sites highly 2′‐O‐methylated in other species may not pertain to mouse embryos (Figure S6F, Supporting Information). However, we only detected marginally reduced 2′‐O‐methylation in rRNAs from *Znhit3^−/−^* embryos at E2.5 and E3.5 at which time control embryos develop into 8‐cells and morulae, respectively (Figure S6G,H, Supporting Information). Thus, we suspect that either ZNHIT3 contributes little to the 2′‐O‐methylation of rRNA or, more likely, that in the absence of ZNHIT3, hypo‐methylated rRNA is quickly degraded. The slightly reduced 2′‐O‐methylation of rRNA after *Znhit3* ablation would contribute very little in the reduced protein translation within *Znhit3* null embryos.

### 2.6 Decreased Abundance of snoRNA, rRNA and Intron Retention After *Znhit3* Ablation

ZNIHT3 is reported to participate in assembly of box C/D snoRNP complex in which a rRNA molecule is recruited to the snoRNP complex by binding to a snoRNA. Thus, we determined if rRNA levels were affected in *Znhit3^−/−^* embryos. Since our sc‐ and se‐RNA‐seq method use poly(A) tails to capture RNA for sequencing. We first wanted to confirm whether the amount of non‐poly(A) RNAs, e.g., rRNA, snoRNA, etc., captured in these sequencing experiments also correlate with their abundances in the embryonic cells. After intercrossing *Znhit3^KI/−^* mice, we collected early morulae and confirmed the genotype of each embryo by an anti‐HA immunofluorescence assay. We then grouped *Znhit3^−/−^* and control embryos, respectively and performed quantitative reverse transcription polymerase chain reaction (qRT‐PCR) after getting their total RNA to examine expression of 18S rRNA in these 2 groups of embryos. Using ERCC spike‐in as the external control during RNA extraction and qRT‐PCR, we could confirm that even for 18S rRNA, a typical non‐poly(A) RNA, the RNA‐seq results correlate well with that from qRT‐PCR, warranting the reliability of our sc‐ and se‐RNA‐seq for quantification of non‐poly(A) RNAs (Figure S5A, Supporting Information). By further analyzing our RNA‐seq results, we confirmed that lower rRNA levels were consistently observed in *Znhit3^−/−^* embryos (compared to control embryos) from E2.5 until E3.5 which corresponds to 8‐cell to morula stage in control embryos (Figure 6E). Similarly, the abundance of many snoRNAs also decreased to low levels in *Znhit3^−/−^* embryos during these developmental stages (Figure 6F,G). Based on these observations, we postulate that ZNHIT3 participates in binding snoRNA to rRNA during box C/D snoRNP complex assembly. Loss of ZNHIT3 reduces this binding and leads to degradation of both snoRNA and rRNA. Reduced rRNA impairs ribosome assembly and function leading to decreased protein translation. Box C/D snoRNP complexes are also reported to participate in nascent mRNA splicing.^[^ ^29^ ^]^ This is confirmed by our IP/MS experiment which identified multiple components responsible for mRNA splicing, e.g., XRN2, DIS3, CNOT8, etc, as another group of binding partners of ZNHIT3 (Table S3, Supporting Information). We also confirmed similar subcellular localization of ZNHIT3 and XRN2 (Figure S5C, Supporting Information). In agreement with these observations, we detected slightly higher amounts of intronic sequences in transcripts captured from *Znhit3^−/−^* embryos at E2.5 and E3.5 in seRNA‐seq experiment. Although the difference is not significant, it suggests ZNHIT3 may be indispensable for proper splicing of nascent mRNAs (Figure 6H). These abnormalities in *Znhit3^−/−^* embryos were also recapitulated in 8‐cell blastomeres (Figure S3C,D, Supporting Information). Taken together, our results indicate that ZNHIT3 participates in recruiting rRNA into the box C/D snoRNP complex which also mediates mRNA splicing. In the absence of ZNHIT3, there is decreased rRNA and abnormalities in pre‐mRNA splicing, both of which could contribute to the impaired protein translation in *Znhit3^−/−^* embryos that leads to embryonic arrest at the morula stage.

### 2.7 Microinjection of cRNA Encoding ZNHIT3 Partially Rescues Embryonic Defects

**Figure 7:**
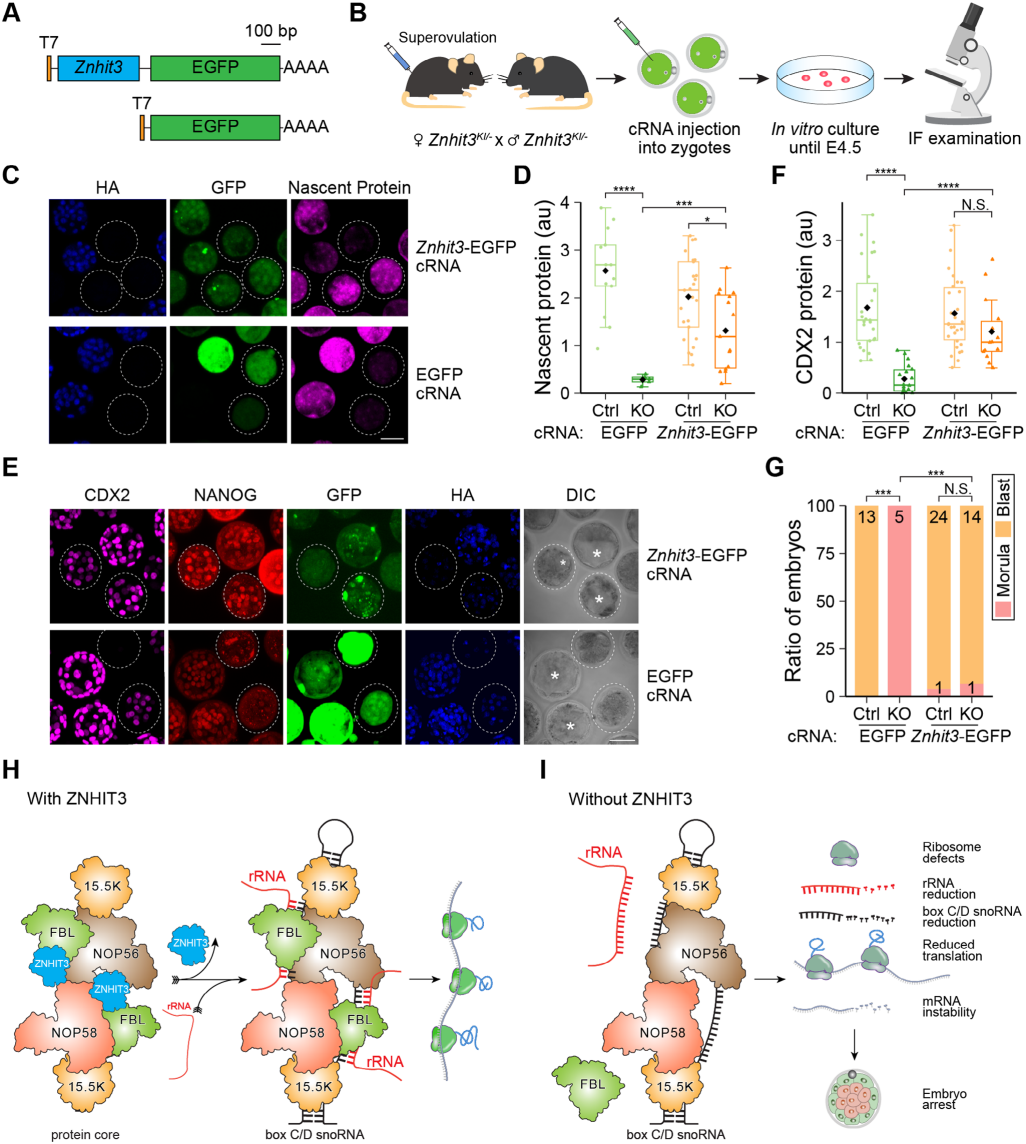
*Znhit3* cRNA partially rescues defects in *Znhit3^−/−^* embryos. A) Schematic of the *Znhit3*‐EGFP cRNA used for microinjection. EGFP cRNA is used as the control. B) Scheme of exogenous cRNA mediated rescue experiment. *Znhit3*‐EGFP or control cRNA are microinjected into late zygotes collected from *Znhit3^KI/−^* female mice after mating with *Znhit3^KI/−^* males. The embryos are cultured in vitro until E4.5 for microscopic examination. C) Images of nascent proteins in *Znhit3^−/−^* (dashed circles) and control (Ctrl) embryos at E4.5 after zygotic injection of EGFP or *Znhit3*‐EGFP cRNA. Scale bar, 50 µm. D) Quantification of nascent protein level in each embryo after injection of different cRNAs. Note *Znhit3* cRNA largely rescues the protein translation in *Znhit3^−/−^* embryos comparing to EGFP cRNA injection. E) Immunofluorescent images of CDX2 and NANOG proteins in *Znhit3^−/−^* (dashed circles) and control embryos at E4.5 after zygotic injection of EGFP or *Znhit3*‐EGFP cRNA. Scale bar, 50 µm. F) Quantification of CDX2 protein level in each embryo after injection of different cRNAs. Note *Znhit3* cRNA largely rescues CDX2 levels in *Znhit3^−/−^* embryos compared to EGFP cRNA injection. It also rescues developmental defects of *Znhit3^−/−^* embryos as they can progress into blastocyst stage. Asterisks in E indicate blastocoels in the embryos and rescued *Znhit3^−/−^* embryos have smaller cavities compared to control embryos, suggesting incomplete rescue. G) Ratio of developmental stages the injected embryos could achieve. The number of embryos captured for each group is labeled on the bars. Note *Znhit3* cRNA rescues the developmental defects of *Znhit3^−/−^* embryos as they can progress into blastocyst stage like control embryos. H) Proposed molecular mechanisms of *Znhit3* in controlling protein translation and early embryo development. ZNHIT3 facilitates assembly of the box C/D snoRNP complex which is essential for rRNA processing and later ribosome functions. This in turn ensures normal protein translation. Without ZNHIT3 I), rRNA cannot be recruited into box C/D snoRNP complex for proper processing which leads to malfunctions of ribosomes, down‐regulation of rRNA and box C/D snoRNA. In combination, these defects reduce protein translation and promotes degradation of noncomplexed RNA in mouse embryos which leads to developmental arrest.

To determine whether the observed defects in preimplantation development could be rescued, we injected EGFP‐conjugated *Znhit3* complementary RNA (cRNA) into late 1‐cell zygotes derived from *Znhit3^KI/−^* female mice after mating with *Znhit3^KI/−^* male mice and cultured them until E4.5 (**Figure** **7**A,B). Adding back ≈1.0 pL of 1.0 µg µL^−1^ *Znhit3* cRNA rescued the morula arrest in all the *Znhit3^−/−^* embryos, which then progressed to blastocysts. In contrast, *Znhit3^−/−^* embryos injected with comparable amounts of 1.0 µg µL^−1^ EGFP cRNA remained as morulae, even though much stronger fluorescence were observed that indicated higher amount of EGFP protein (Figure 7C,E; Figure S7A,C,G, Supporting Information). Protein translation was largely recovered in *Znhit3^−/−^* embryos supplemented with *Znhit3* cRNA, but not with EGFP control cRNA, as confirmed by immunofluorescence of nascent protein (Figure 7C,D; Figure S7A, Supporting Information). We further confirmed that the nascent protein level was positively correlated with expression of *Znhit3* cRNA (Figure 7C; Figure S7B, Supporting Information). Similarly, we also observed recovered expression of NANOG and CDX2 in *Znhit3^−/−^* embryos injected with *Znhit3* cRNA (Figure 7E,F; Figure S7C,D, Supporting Information) However, exogenous injected *Znhit3* cRNA failed to increase protein translation as well as the NANOG and CDX2 abundance to the same extent as control embryos (Figure 7C‐F; Figure S7A,C,D, Supporting Information). This may be due to insufficient accumulation of ZNHIT3 protein at E4.5 as significant decrease of ZNHIT3‐EGFP protein was observed during embryo development after injection. In agreement with this observation, although *Znhit3^−/−^* embryos progressed to the blastocyst stage after zygotic injection of *Znhit3* cRNA, these embryos had fewer blastomeres, smaller blastocoels and smaller size compared to control embryos (Figure 7E; Figure S7E‐G, Supporting Information). Nevertheless, these results indicate that ZINHIT3 is indeed a key component in controlling protein translation in early embryos, absence of which is sufficient to cause translation defects and morula arrest.

## 3 Discussion

Early mammalian embryo development requires reprogramming the zygotic epigenome and differentiation of cells to establish germ layers.^[^ ^30^ ^]^ Both processes occur quickly and require rapid accumulation of proteins that define identity and execute cell function. eIF4E1b, an oocyte‐specific translation initiation factor,^[^ ^31^ ^]^ selects and stabilizes essential maternal mRNAs for translation of key factors responsible for reprogramming the zygotic epigenome to ensure the maternal to zygotic transition.^[^ ^8^ ^]^ After zygotic activation, the rapid expression of essential genes establishes protein programs to ensure progression of embryonic cells into different lineages. This process is accompanied by increased translation efficiency^[^ ^10^, ^12^ ^]^ and we now report that ZNHIT3 regulates protein translation to ensure the proper development of mouse embryos.

ZNHIT3 was previously identified as a co‐factor that participates in box C/D snoRNP complex assembly^[^ ^17^, ^32^ ^]^ but its molecular role in preimplantation mammalian development remained unexplored. By anti‐ZNHIT3^FLAG^ IP followed by mass spectrometry, we identified multiple core components of the box C/D snoRNP complex including NOP58, NUFIP1, FBL, etc. Together with immunofluorescence experiments, we confirm that ZNHIT3 is indeed another key component of box C/D snoRNP complex in embryonic cells (Table S3 and Figure S5B, Supporting information). Fibrillarin (FBL) is a core component of box C/D snoRNP complex that is essential for early embryo development.^[^ ^15^ ^]^ However, *Fbl* transcript remains largely unchanged in blastomeres during early embryo development (Figure S1A, Supporting Information) suggesting a structural rather than regulatory role. By establishing gene‐edited mice in which ZNHIT3 is tagged with FLAG and HA (*Znhit3^KI^*), we document that the protein is first detected in mid 2‐cell embryos and is abundant throughout preimplantation development (Figure 1C,D). These results agree with previously published transcriptome and proteome results and are consistent with *Znhit3* being a single‐copy zygotic gene. Its increased expression in later stages of preimplantation development also provides evidence for a role as a facilitator of box C/D snoRNP function at which time protein translation should be promoted.

*Znhit3* null embryos arrest at the morula stage of development due to impaired protein translation with a secondary effect that decreases abundance of unprotected transcripts.^[^ ^33^ ^]^ The first cell lineage dichotomy become detectable when mouse embryos progress to morulae.^[^ ^9^, ^24^, ^30^ ^]^ Blastomeres with high expression of pluripotency factors, e.g., NANOG, OCT4, meanwhile represses pathways controlled by CDX2. These cells later become the inner cell mass (ICM) which ultimately give rise to all the cells of the animal. In contrast, morula blastomeres with high CDX2 and low abundance of pluripotency factors contribute to the trophectoderm that facilitates the embryo implantation and development. In embryos with ZNHIT3 depletion, expression of these two opposing transcription factor groups both decrease (Figure 5) which may compromise cell fate commitment programs and lead to morula arrest. The impaired protein translation machinery subsequently correlates with degradation of these arrested embryos (Figure 2E‐H). By injecting cRNA encoding ZNHIT3 into *Znhit3^−/−^* late zygotes, we successfully rescued translation in these embryos as well as abundance of NANOG and CDX2 (Figure 7A‐F). However, the rescued embryos did not recapitulate all the features of control embryos as they had fewer blastomeres and smaller size (Figure 7E; Figure S7E‐G, Supporting Information). It may be that the turnover of the microinjected cRNA in *Znhit3^−/−^* zygotes and the continuing need for translation precludes a complete rescue.

One function of box C/D snoRNP complex is to regulate 2′‐O‐methylation of rRNA. Using SMARM‐seq, we successfully profiled rRNA 2′‐O‐methylation in groups of embryos, but after *Znhit3* ablation we observed only a marginal decrease of rRNA 2′‐O‐methylation suggesting that ZNHIT3 may not direct 2′‐O‐methylation in the mutant mice. Nevertheless, our newly developed SMARM‐seq protocol shows similar 2′‐O‐methylation pattern for many well‐known rRNA residues, indicating its reliability while using a much smaller amount of materials (Figure S6C,D, Supporting Information). Both rRNA and box C/D snoRNA levels were reduced in *Znhit3^−/−^* embryos indicating that ZNHIT3 facilitates recruitment of rRNA into the box C/D snoRNP complex. RNAs (rRNA and snoRNA) not recruited into the complex are rapidly degraded due to lack of protection by complexing proteins.^[^ ^34^ ^]^ Thus, we could not exclude the possibility that ZNHIT3 depletion indeed reduces 2′‐O‐methylation of some rRNAs that could not be assembled into the snoRNP complex, but our method failed to capture this methylation change due to rapid degradation of these unprotected rRNAs. Because active translation has been documented to prevent mRNA degradation,^[^ ^33^, ^35^ ^]^ it is not surprising that extensive decrease of mRNA is observed in *Znhit3^−/−^* embryos. Many snoRNAs also participate in the pre‐mRNA splicing.^[^ ^36^ ^]^ We also identified many components responsible for pre‐mRNA splicing as another group of binding partners of ZNHIT3 in the IP/MS experiment (Table S3 and Figure S5C, Supporting Information). This also suggests ZNHIT3 to be a mediator for box C/D snoRNP complex in recruiting pre‐mRNA splicing machineries. It is thus not surprising that ZNHIT3 deletion leads slightly increased intron retention which could be another reason for the mRNA degradation as non‐properly processed mRNAs are destined for clearance.^[^ ^37^ ^]^ The combined defects that halt the protein translation in *Znhit3^−/−^* embryos arrest development at the morula stage of preimplantation development (Figure 7H,I).

In this paper, we illustrate how ZNHIT3 plays a key role to ensure the progression of early mouse embryogenesis by affecting box C/D sonRNP complex assembly and protein translation. Future investigations into box C/D snoRNP function could use ZNHIT3 as a key point to explore new regulatory mechanisms during the early embryo development. Our results could also guide research in human disease conditions as *Znhit3* mutations have been reported to cause progressive encephalopathy with oedema, hypsarrhythmia, and optic atrophy (PEHO) syndrome.^[^ ^38^ ^]^ It would be useful to explore how protein synthesis may be affected under the pathological condition caused by *Znhit3* mutations and hopefully promoting protein translation could be a therapeutic modality for human patients with defective *Znhit3*.

## 4 Experimental Section

### Establishment of Mouse Lines—Generation of CRISPR/Cas9 Mutant Mice

All animal studies were performed in accordance with guidelines of the Animal Care and Use Committee of the National Institutes of Health under Division of Intramural Research, National Institute of Diabetes and Digestive and Kidney Diseases‐approved animal study protocols (K018‐LCDB‐24 and K044‐LCDB‐22). Two CRISPR‐Cas9 crRNA XT oligonucleotides^[^ ^39^ ^]^ were used to establish the *Znhit3^KO^* mutant line. 1.5 µL of each crRNA‐tracrRNA duplex solution (100 mM) after annealing crRNA to tracrRNA (1072533, Integrated DNA Technologies [IDT]) was mixed and used for electroporation. Another crRNA XT oligonucleotide as well as a single strand DNA (ssDNA) oligo were used to establish the mouse line containing FLAG and HA tags fused at the carboxyl terminus of ZNHIT3. Detailed protocols were described previously.^[^ ^8b^ ^]^ All crRNA XT oligonucleotides and the ssDNA for repair were synthesized by Integrated DNA Technologies (IDT). Sequences for the sgRNA and repair oligonucleotides are provided in Table S5 (Supporting Information).

### Establishment of Mouse Lines—*Genotyping*

Tail tips of mice were lysed in 200 µL of DirectPCR Lysis Reagent (102‐T, Viagen Biotech) with proteinase K (3115879001, 0.2 mg/ml, Sigma–Aldrich) at 55 °C for 4–16 h. To inactivate proteinase K, samples were incubated at 85 °C for 1 h. EmeraldAmp GT PCR Master Mix (RR310A, Takara Bio USA) and gene specific primers (Table S5, Supporting Information) were used to amplify specific DNA fragments. PCR was performed with an annealing temperature of 59 °C and 37 cycles using Mastercycler Pro (Eppendorf). For single embryo genotyping, single morula or blastocyst was lysed in 4 µL 1× Q5 PCR buffer containing high GC enhancer (B9027S, New England Biolabs [NEB]) and proteinase K (3115879001, 0.2 µg µL^−1^, Sigma–Aldrich). 56 °C 30 min, 95 °C 15 min and use directly for genotyping PCR.

### Analysis of Single Embryos—Immunofluorescence of Mouse Embryos

Mouse embryos were fixed in 4% paraformaldehyde (PFA; 15710, Electron Microscopy Sciences) for 30 min at room temperature and washed in phosphate‐buffered saline (PBS; 10010023, Invitrogen) supplemented with 0.3% polyvinylpyrrolidone (PVP; PVP360‐100G, Sigma–Aldrich). Eggs/embryos were incubated in PBS with 0.3% bovine serum albumin (BSA; 9998S, Cell Signaling Technology [CST]) and 0.1% Tween 20 (P9416, Sigma–Aldrich) for 2 h and stained overnight at 4 °C with primary antibodies. Goat anti‐mouse or rabbit antibody conjugated with Alexa Fluor was used for immunofluorescent imaging. Primary antibodies used were anti‐HA (3724S, 1:600, CST), anti‐HA (NBP2‐50416, 1:300, Novus Biologicals), anti‐OCT4 (sc‐5279, 1:60, Santa Cruz Biotechnology), anti‐CDX2 (Ab76541, 1:200, Abcam), anti‐NANOG (sc‐374103, 1:80, Santa Cruz Biotechnology), anti‐Phospho‐S6 Ribosomal Protein (Ser235/236) (2211S, 1:100, CST), anti‐NUFIP1(12515‐AP, 1:200, Proteintech) and anti‐XRN2 (IHC‐00223, 1:200, Berthyl Laboratories). For anti‐OCT4 and anti‐NANOG immunostaining, non‐specific signals from zonae pellucidae were computationally removed with a home‐made MATLAB script before quantification of the intensity. All the experiments were repeated at least three times and representative results from one replicate were presented.

### Analysis of Single Embryos—Count of Blastomere Numbers in Single Embryos

Immunofluorescent (IF) Z‐stack data were used to determine the number of blastomeres in each embryo after staining with DAPI. The Z‐stack data for one embryo were first split into images reflecting each layer. To determine the number of blastomeres in an embryo, a MATLAB script was used to examine the DAPI staining for each image representing distinct layers of the IF experiment. After examining all layers of images, each blastomere was labeled and all blastomeres were projected into a single image. The position of each blastomere was plotted in the projected image and the total number of blastomeres was determined.

### Single Embryo RNA‐seq (seRNA‐seq)

Single embryo RNA‐seq was adapted from single cell G&T‐seq.^[^ ^23^ ^]^ To collect embryos, fertilized zygotes were flushed from hormone simulated *Znhit3^+/−^* female mice after successful mating with *Znhit3^+/−^* males and cultured in advanced KSOM medium (MR‐101‐D, Millipore) until sample collection. When collecting samples, embryos were washed in PBS and transferred into acidic Tyrode's solution (MR‐004‐D, Millipore) to remove zonae pellucidae. Single zona‐free embryos were transferred into 8‐well PCR strips containing 2.5 µL of RLT Plus buffer (1053393, Qiagen Sciences). The PCR strips with single embryos were frozen at ‐80 °C until library construction. The G&T‐seq protocol was used to generate seRNA‐seq libraries. During RNA‐seq library construction, 1 µL of pre‐diluted (1:10^5^) ERCC spike‐in (4456740, ThermoFisher Scientific) was added to each well containing a single embryo and 15 PCR cycles were used for cDNA amplification. Indexed single embryo RNA‐seq libraries were pooled and purified with AMPure XP beads (A63881, Beckman Coulter) at a ratio of 1:0.6. The quality of the pooled libraries was confirmed by Bioanalyzer 2100, and each pooled library was sequenced (51 bp, paired‐end) in one lane on the Illumina HiSeq2000 platform in the NIDDK Genomics Core, or (150 bp, paired‐end) in one lane on the Illumina HiSeq4000 platform (Novogene).

### Low Input Small RNA Smart‐seq Facilitated RiboMeth‐seq (SMARM‐seq)

*Znhit3^KI/−^* female mice were hormone simulated (5 IU eCG followed 48 h later with 5 IU hCG, both intraperitoneal) and co‐caged with *Znhit3^KI/−^* males for collection of zygotes. Zygotes without cumulus were collected as previously described and cultured until the desired developmental stages. Before sample collection, embryos were first examined by a rapid immunofluorescence (IF) staining to confirm expression of the HA tag fused to ZNHIT3 in the knock‐in (KI) allele. The IF was performed with 1 h incubation of primary antibody and 0.5 h of secondary antibody incubation at room temperature. All IF reagents were supplied with 0.5U µL^−1^ RNase inhibitor. Embryos without the knock‐in allele were considered homozygous null and others as controls. 15–20 zona‐free homozygous null or control embryos were used as one group which were washed in PBS and then transferred into acidic Tyrode's solution to remove zonae pellucidae before collecting into PCR strips with 5 µL RLT Plus lysis buffer. 9 µL RNAclean beads (1:1.8 ratio) were used to purify RNA in each group. 10 µL 50 mM Na_2_CO_3_/NaHCO_3_ buffer (pH 9.2) were used for elution with 5 min incubation before heating at 95 °C for 10 min in a PCR cycler to finish hydrolysis. 1 µL 1N HCl was added to neutralize the pH. 22 µL RNAclean XP beads and 28 µL 100% isopropanol were then added to bind the fragmented RNA in each group. The beads were washed twice with 180 µL 85% ethanol and eluted with 10 µL Antarctic phosphatase reaction mix containing 7.5 µL H_2_O, 1 µL 10x Antarctic phosphatase reaction buffer, 1 µL Antarctic phosphatase (M0289S, New England Biolabs [NEB]) and 0.5 µL 20U µL^−1^ RNase inhibitor. Incubation at 37 °C for 30 min and then 70 °C for 5 min was performed to finish de‐phosphorylation and enzyme inactivation. After de‐phosphorylation, RNA fragments were purified by adding 18 µL RNAclean XP beads and 27 µL 100% isopropanol. The RNAclean XP beads were eluted with 10 µL poly‐adenylation mix from the SMARTer smRNA‐Seq kit (635029, Takara) after washing. Other steps of the product manual were followed to finish the library preparation with 14 PCR cycles for library amplification. Sequenced reads of SMARM‐seq libraries were trimmed with Cutadapt ver. 4.2 using AAAAAAAAAA as the adapter sequence.^[^ ^40^ ^]^ Trimmed reads were aligned to mouse rDNA sequence (GenBank: BK000964.3)^[^ ^41^ ^]^ with RNA‐STAR^[^ ^42^ ^]^ and PCR duplicated were removed from the aligned BAM file by Picard in GATK ver. 4.3.0.0.^[^ ^43^ ^]^ SAMtools^[^ ^44^ ^]^ was used to get SAM files from the BAMs without PCR duplicates which were examined by a home‐made C++ program to check the rRNA base at the 5′ end of each sequencing read. The frequencies of rRNA bases detected to be the 5′ end of sequencing reads was used to calculate the RiboMeth score based on previously published algorithm.^[^ ^27^, ^45^ ^]^

### Analysis of Blastomeres—Single Cell RNA‐seq (scRNA‐seq)

Single zona‐free embryos were first collected as described above. After that, one single embryo was pipetted into a capillary tube with a diameter of 1.5 to 2 times of the diameter of single blastomeres to physically dissociate single blastomeres. Single blastomeres that remained intact were transferred into PCR strips and processed using the G&T‐seq to finish the RNA‐seq library. 1 µL of 1:10^6^ diluted ERCC was added to lysate of each single blastomeres from 8‐cell embryos, 17 PCR cycles were used to amplify the cDNA.

### Analysis of Blastomeres—Analysis of seRNA‐seq and scRNA‐seq Data

Alignment of se/scRNA‐seq reads as well as sample filtration and data analysis were described previously.^[^ ^8b^ ^]^ 86 of the 94 single embryos for seRNA‐seq and 82 of the 96 single 8‐cell blastomeres for scRNA‐seq were used for downstream analysis. Number of reads that aligned to the *Znhit3* deleted regions in *Znhit3^−/−^* line was used to confirm the genotype of each sample. DESeq2 was used to analyze the cleaned RNA‐seq data^[^ ^46^ ^]^ for samples from the same developmental stages. The ERCC normalized gene count matrix was used for all plots. PCA and MA plots were generated using R. The gene biotype information was downloaded from Biomart.^[^ ^47^ ^]^

### Analysis of Blastomeres—Analysis of rRNA Expression from scRNA‐seq or seRNA‐seq

Alignment and analysis for rRNA expression follows similar procedures as described in the previous scRNA‐seq analyses. Briefly, the FASTQ reads that cannot align to the annotation file in sc/seRNA‐seq were then re‐aligned by STAR against rRNA annotations generated from BK000964.3. Only uniquely mapped reads from the BAM file generated by STAR were kept and Stringtie^[^ ^48^ ^]^ was used to calculate expression of all rRNAs.

### Analysis of Intron and Exon Sequences from RNA‐Seq Experiments

After alignment of sc‐ or se‐RNA‐seq FASTQ files to obtain the correlated BAM files, the exon count of each transcript in each sample was calculated by featureCounts v2.0.3.^[^ ^49^ ^]^ A home‐made C++ program was used to calculate the intron counts using the GTF annotation and SAMtools transformed SAM files from the BAM files. Only reads containing more than 3 continuous intron bases were considered an effective intron reads.

### Quantitative Reverse Transcription Polymerase Chain Reaction (qRT‐PCR) of 18S rRNA

Morulae were collected after intercross of *Znhit3^KI/−^* mice. A rapid anti‐HA IF was performed by reducing the BSA blocking time to 0.5 h and primary antibody incubation for 1 h, both at room temperature in the presence of RNase inhibitor. After examining the anti‐HA IF results, *Znhit3^−/−^* embryos were group together with minimal buffer and lysed in RLT Plus buffer. About 20 *Znhit3^−/−^* embryos were grouped together while ≈20 control embryos were used as another group. ERCC spike‐in was then added to the grouped and lysed embryos. ERCC was diluted in RLT Plus buffer by 1:10^3^ and 1 µL diluted ERCC was added for one embryo. The lysis was then used for total RNA extraction using RNeasy Plus Micro Kit (74034, Qiagen) followed by reverse transcription using the SuperScript IV VILO Master Mix (11756050, ThermoFisher Scientific). qRT‐PCR were then performed to examine 18S rRNA level in the embryos with ERCC as external control. Primers used for qRT‐PCR are listed in Table S5 (Supporting Information).

### Analysis of Proteins—Analysis of ZNHIT3 Protein Sequences

To generate the phylogenetic tree using ZNHIT3 protein sequences from different vertebrates, corresponding protein sequences of selected vertebrates were downloaded from NCBI and then analyzed by MEGA 11.^[^ ^21^ ^]^ The maximum likelihood evolutionary analysis method provided in MEGA 11 was used for the analysis.^[^ ^50^ ^]^ The accession numbers of protein sequences analyzed are: NP_001005223.1 (mouse), NP_956567.1 (zebrafish), NP_001091379.1 (frog), XP_030392805.1 (turtle), XP_015151640.2 (chicken), XP_003639345.1 (dog), NP_001385779.1 (rat), XP_005076970.1 (hamster), XP_003912918.1 (baboon), XP_004041917.1 (gorilla), NP_004764.1 (human). The ZNHIT3 protein sequences of mouse, rat, dog and human were further aligned with Clustal X.^[^ ^22^ ^]^

### Analysis of Proteins—Anti‐ZNHIT3^FLAG^ Immunoprecipitation Followed by Mass Spectrometry (IP/MS)

ZNHIT3 protein with C terminal FLAG tag was expressed in mouse embryonic stem cells E14, after transfection the cells with a pCAG plasmid containing the expression cassette. After transfection (48 h), one 10 cm dish was used to harvest the cells in RIPA buffer (89900, ThermoFisher Scientific) for anti‐FLAG IP with M2 magnetic beads (M8823, Sigma). E14 cells transfected with the plasmid backbone were used as the control. The M2 beads after IP were sent to National Institute of Diabetes and Digestive and Kidney Diseases (NIDDK) Advanced Mass Spectrometry Core for mass spectrometry analysis. The full gene list encoding possible ZNHIT3 binding partners are provided in Table S3 (Supporting Information).

### Analysis of Proteins—Embryo Treatment and Imaging of Protein Synthesis

To determine differences of mRNA translation in embryos with different genotypes, zygotes were obtained from hormonally simulated *Znhit3^KI/−^* females after intercross with *Znhit3^KI/−^* males and cultured in advanced KSOM medium until the time of treatment. The Click‐iT Plus OPP (O‐propargyl‐puromycin) Alexa Fluor 488 Protein Synthesis Assay Kit (C10456, ThermoFisher Scientific) was used to determine nascent protein synthesis in each group of embryos. Embryos not stained by anti‐HA antibody were designated as *Znhit3^−/−^* and others were used as controls. Nuclei were labeled with DAPI. All the experiments were repeated at least three times and representative results from one replicate were presented.

### Analysis of Proteins—Quantification of Fluorescence Intensity

For all fluorescent staining experiments, the fluorescence intensity in each egg/embryo was quantified by ImageJ version 1.53k^[^ ^51^ ^]^ and then used for plotting in R.

### mRNA In Vitro Transcription and Microinjection

The In‐Fusion cloning method was used to clone mouse *Znhit3* cDNA into pCDNA3.1+ plasmid with EGFP sequence fused at its C terminus. The pCDNA3.1+ plasmid with only EGFP was used as control. These plasmids were then used to prepare corresponding cRNAs for microinjection into mouse late zygotes as previously described.^[^ ^8^, ^16^ ^]^ Approximately 1.0 pL of cRNA solution was injected into each zygote. A MATLAB script was used for calculation of embryo size after cRNA injection. The projected EGFP immunofluorescent (IF) Z‐stack images were used to determine the size of each embryo. The zona pellucida area was excluded in calculation of embryo size.

### Statistical Analysis

All box plots include the median (horizontal line) and data between the 25th and 75th percentile and each dot reflects one value detected. The black or white diamond within each box shows average within each group. When gene counts from RNA‐seq results in this study were used for plots, they were first normalized by ERCC spike‐in before plotting. The two‐tailed Student's t‐test was used to calculate R values from data of the box plots and bar plots, as well as χ2 test in Figure 7G: N.S. not significant, * P < 0.05, ** P < 0.01, *** P < 0.001, **** P < 0.0001.

## Conflict of Interest

The authors declare no conflict of interest.

## Author Contributions

G.Y. and Q.X. contributed equally to this work. G.Y., Q.X. and J.D. conceived the project. G.Y., Q.X. and J.D. designed the experiments. G.Y. and Q.X. performed the experiments. G.Y. analyzed results and wrote the manuscript with input from X.Q. J.D. revised the manuscript. All authors discussed and approved the manuscript.

## Supporting information



Supporting Information

Supplemental Table 1

Supplemental Table 2

Supplemental Table 3

Supplemental Table 4

Supplemental Table 5

Supporting Information

## Data Availability

The data that support the findings of this study are available from the corresponding author upon reasonable request.
