# Supplementary material for: ZNHIT3 Regulates Translation to Ensure Cell Lineage Differentiation in Mouse Preimplantation Development
Source: Adv Sci (Weinh). 2025 Apr 3;12(21):2413599. doi: 10.1002/advs.202413599 (PMC12140352; doi:10.1002/advs.202413599)
Supplement: Supplementary file 1 — Supporting Information [file ADVS-12-2413599-s005.docx]

**Supporting Information**

**ZNHIT3 REGULATES TRANSLATION TO ENSURE CELL LINEAGE DIFFERENTIATION IN MOUSE PREIMPLANTATION DEVELOPMENT**

Guanghui Yang^1,2^, Qiliang Xin^2*^ and Jurrien Dean^*^

Laboratory of Cellular and Developmental Biology, NIDDK

National Institutes of Health, Bethesda, MD 20892, USA

^1^Present addresses: Howard Hughes Medical Institute, Department of Biology, The Johns Hopkins University, 3400 North Charles Street, Baltimore, MD 21218, USA

^2^These authors contributed equally to this work

^*^Correspondence: [jurrien.dean@nih.gov](mailto:jurrien.dean@nih.gov) (J.D.), [qiliang.xin@nih.gov](mailto:qiliang.xin@nih.gov) (Q.X.)

**This PDF file includes:**

Figures S1 to S7.

**Other Supplemental Materials for this manuscript include the following:**

Tables S1 to S5; Data S1


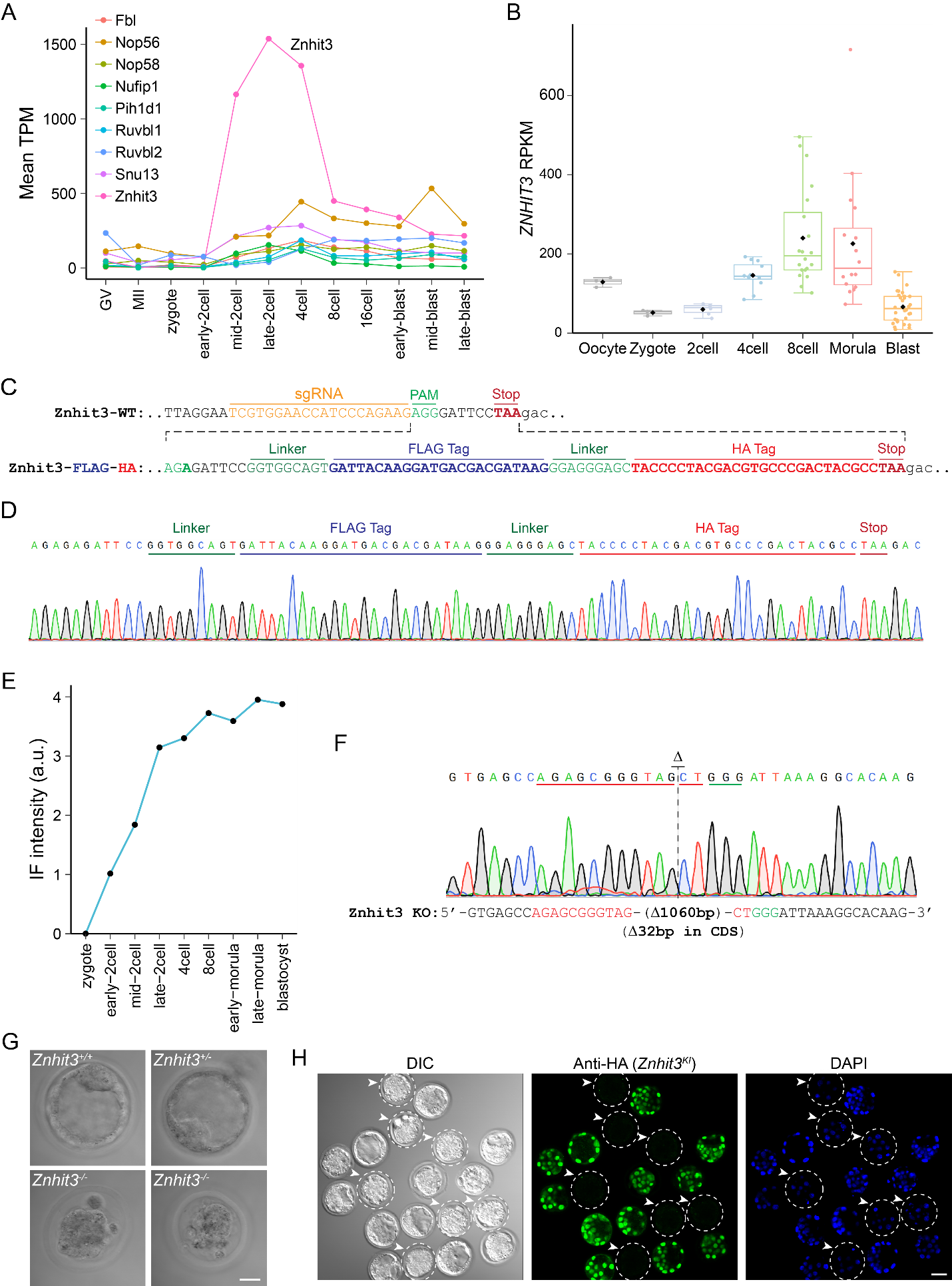


**Figure S1.** Design and confirmation of the *Znhit3* gene-edited mouse lines. A) Published scRNA-seq results of mouse pre-implantation embryos were used to calculate the average abundance of each mRNA in single embryonic cells at different developmental stages.^[16]^ B) Abundance of *ZNHIT3* mRNA from published scRNA-seq results using human pre-implantation embryos.^[18]^ Note the *ZNHIT3* mRNA level greatly increased at the 8-cell stage at which time the human zygotic genome begins to activate. C) Design of the *Znhit3^KI^* mouse line with FLAG and HA tags fused to C-terminus of the protein. D) DNA sequencing at the *Znhit3^KI^* locus of the knock-in mouse line confirmed the tag insertions. E) Quantification of total amount of ZNHIT3 protein levels in each embryo during preimplantation development as determined by anti-HA immunofluorescence in Figure 1D. F) Genomic sequences at the *Znhit3* locus in the *Znhit3^KO^* mouse line with a deletion of 1060 bp (32 bp in the protein coding sequence). G) Representative images of *in vivo* recovered embryos after intercross of *Znhit3^+/-^* mice at E4.0. Scale bar, 20 μm. H) Representative images of *in vivo* recovered embryos after intercross of *Znhit3^KI/-^* mice at E3.5. Control embryos have progressed to the blastocyst stage while *Znhit3^-/-^* embryos (arrowheads, dashed circles) are still morulae with fewer than 32 blastomeres. Scale bar, 40 μm.

**
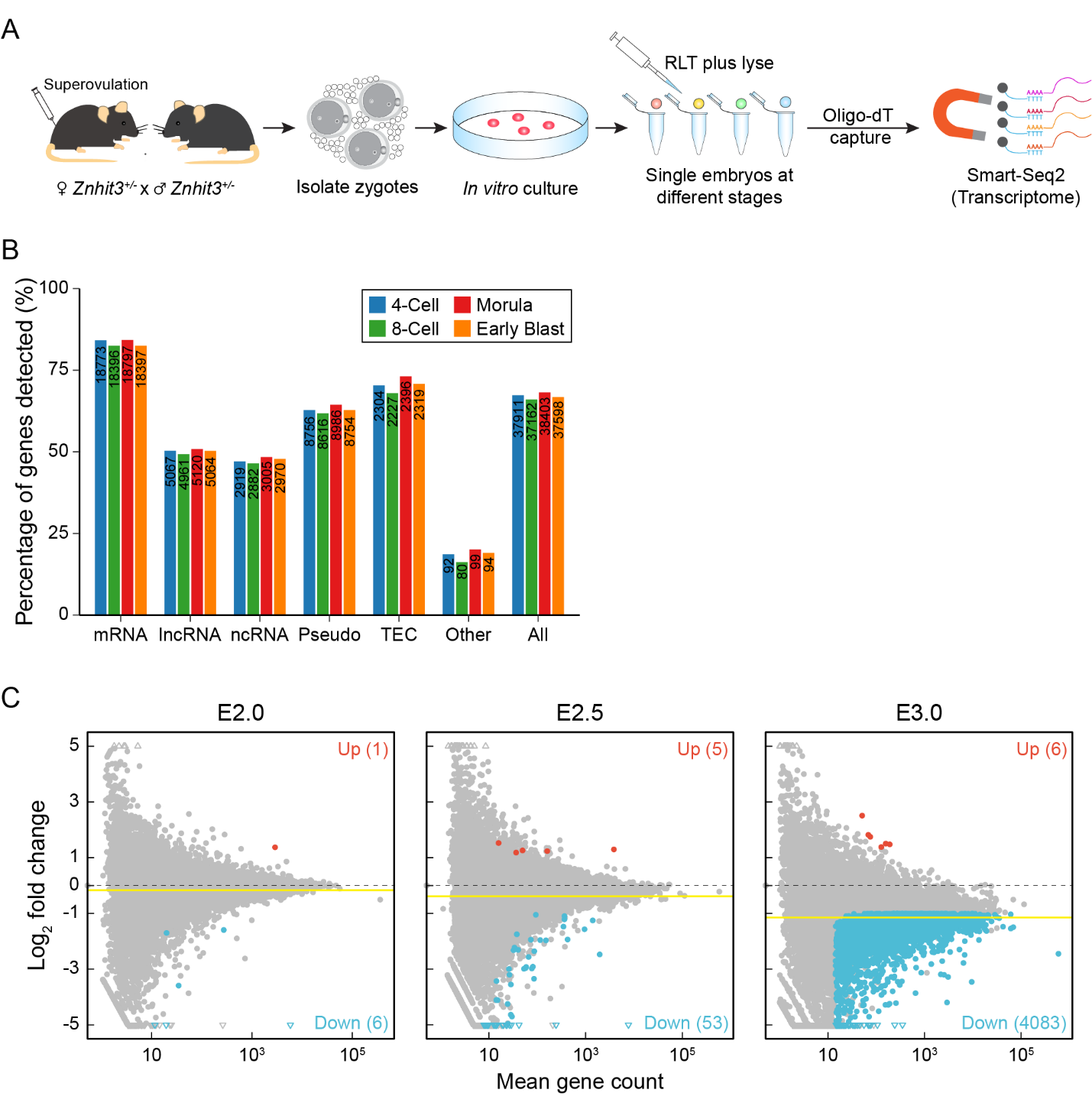
Figure S2.** Design and analysis of seRNA-seq. A) Scheme of single-embryo (se) RNA-seq. *Znhit3^+/-^* female mice were hormonally stimulated and mated with *Znhit3^+/-^* males. Zygotes were collected and cultured *in vitro* until the desired stage of development. Oligo-dT magnetic beads were used to capture RNA in the single embryo lysis and processed following the Smart-Seq2 protocol to obtain transcriptome profiles of single embryos. B) Biotypes of RNAs detected by seRNA-seq. Protein, mRNA; lncRNA, long non-coding RNA; ncRNA, non-coding RNA; Pseudo, pseudogene RNA; TEC, to be experimentally confirmed. C) MA plots document differentially expressed genes between *Znhit3^-/-^* and control embryos. Genes with significantly increased and decreased transcripts are shown as red and blue dots, respectively. The total number of differentially expressed genes is labelled in each plot. The yellow lines indicate the average values of log_2_ fold changes which show global down regulation of genes in *Znhit3^-/-^* embryos.

**
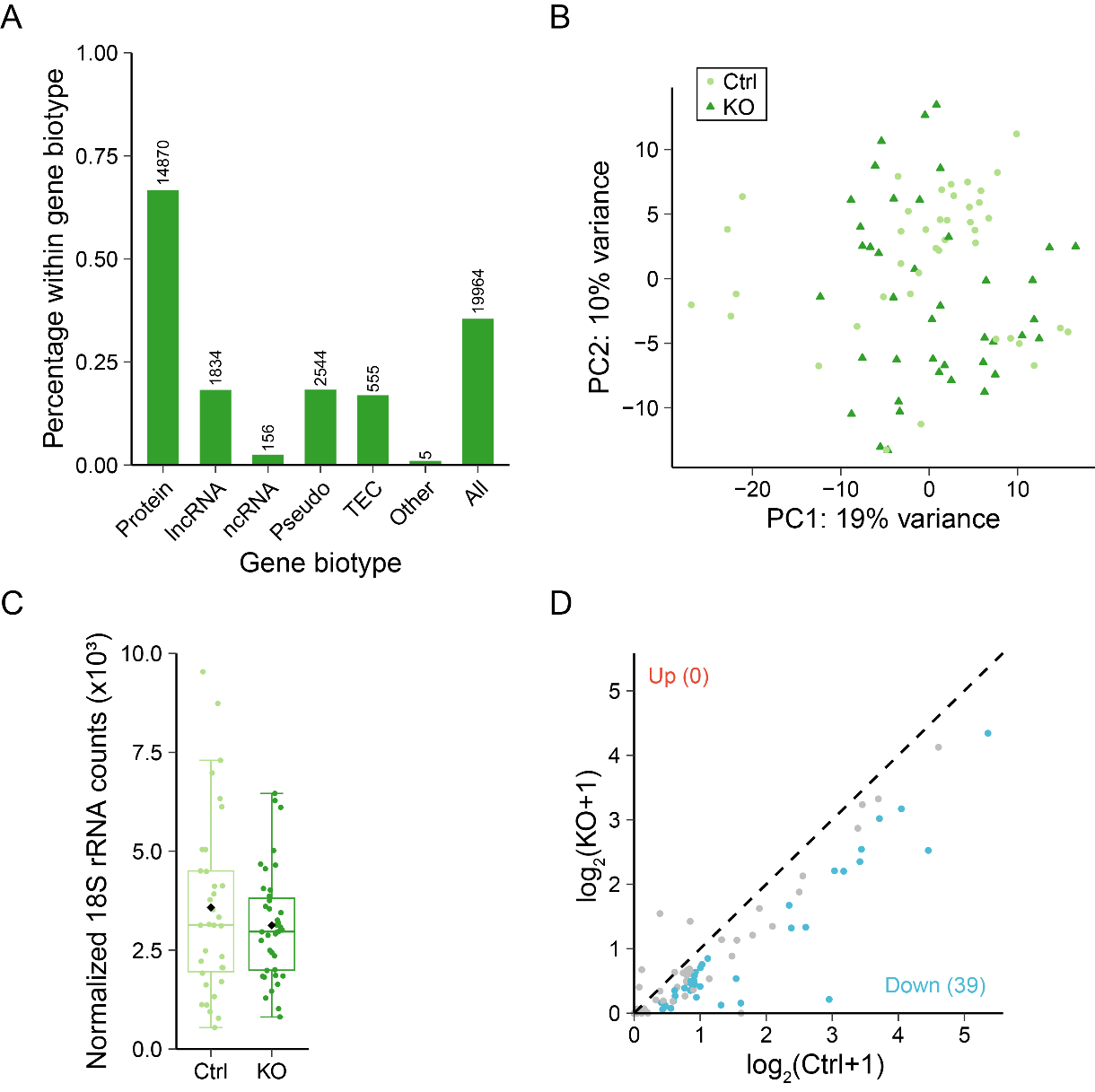
**

**Figure S3.** Blastomeres in *Znhit3^-/-^* 8-cell embryos have abnormalities related to protein translation. A) Biotypes of RNAs detected by single-cell (sc) RNA-seq. Protein, mRNA; lncRNA, long non-coding RNA; ncRNA, non-coding RNA; Pseudo, pseudogene RNA; TEC, to be experimentally confirmed. B) Principal component analysis (PCA) plot of scRNA-seq results in *Znhit3^-/-^* or control blastomeres from 8-cell embryos. At this developmental stage, the *Znhit3^-/-^* blastomeres are not distinguishable from controls. C) Abundance of 18S rRNA in blastomeres from *Znhit3^-/-^* and control embryos at 8-cell stage. D). Scatter plot showing abundance of box C/D snoRNAs in blastomeres from *Znhit3^-/-^* and control embryos at 8-cell stage. The box C/D snoRNAs that are significantly down regulated in the *Znhit3^-/-^* samples were labeled in blue with their total number specified. No box C/D snoRNAs were significantly up regulated in the *Znhit3^-/-^* samples.

**
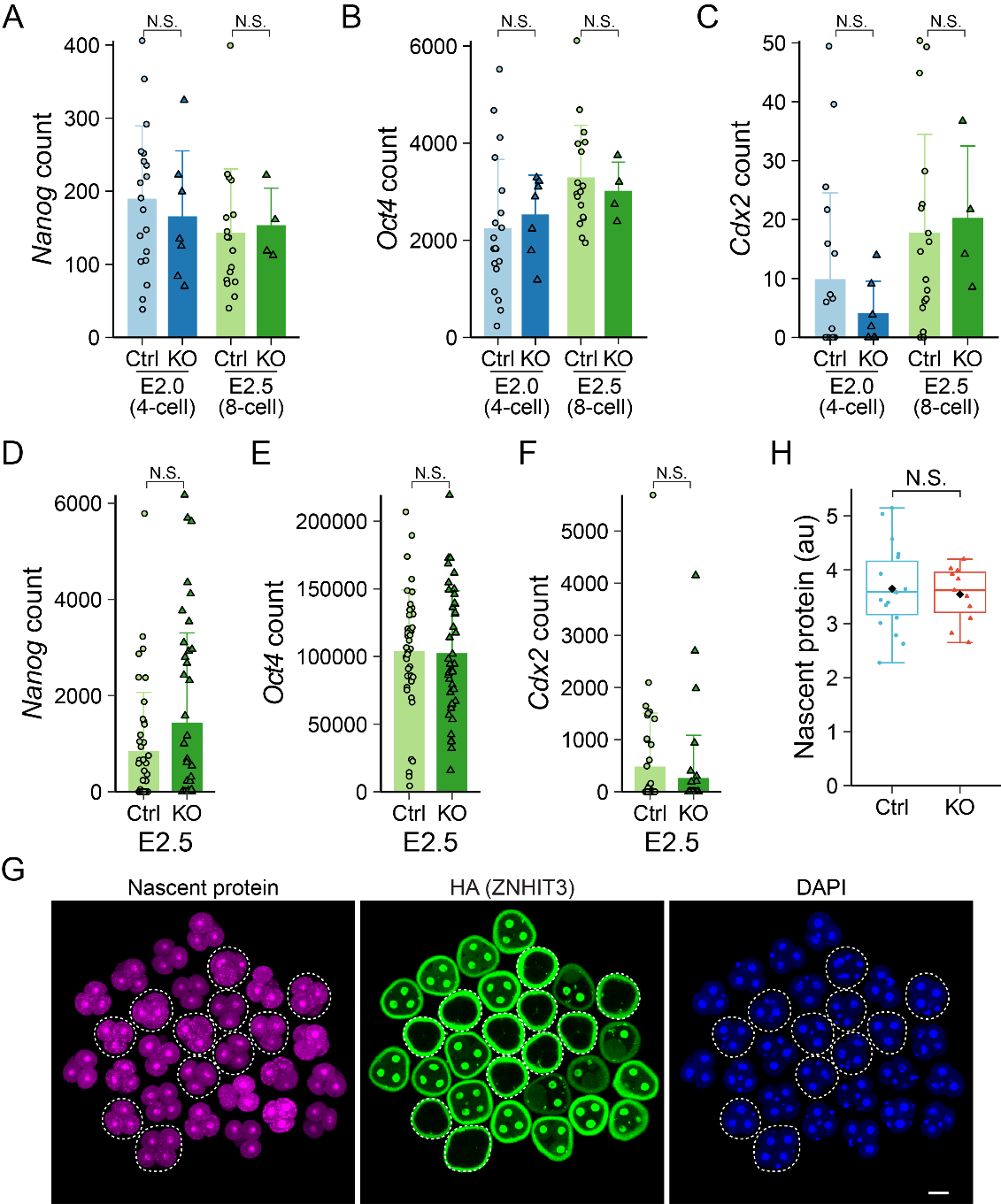
**

**Figure S4.** Expression of transcription factors controlling the first cell fate commitment in mouse morulae. A,B) Expression levels of *Nanog*, *Oct4*, two pluripotent genes that promote the inner cell mass lineage during the first cell differentiation in mouse morula embryos, in *Znhit3^-/-^* (KO) and control (Ctrl) embryos at 4- and 8-cell stages as determined in seRNA-seq. ERCC spike-ins are used to normalize the read counts. C) Same as A,B but for expression of *Cdx2*, a marker gene of trophoblast lineage during the first cell fate commitment. D-F) Expression levels of *Nanog*, *Oct4* and *Cdx2* genes in 8-cell blastomeres as determined by scRNA-seq. G) Images of nascent proteins in *Znhit3^-/-^* (dashed circles) and control (Ctrl) embryos at 4-cell stage. The fluorescent signals are quantified in H and no significant differences are observed between *Znhit3^-/-^* and control embryos. Scale bar, 40 μm.


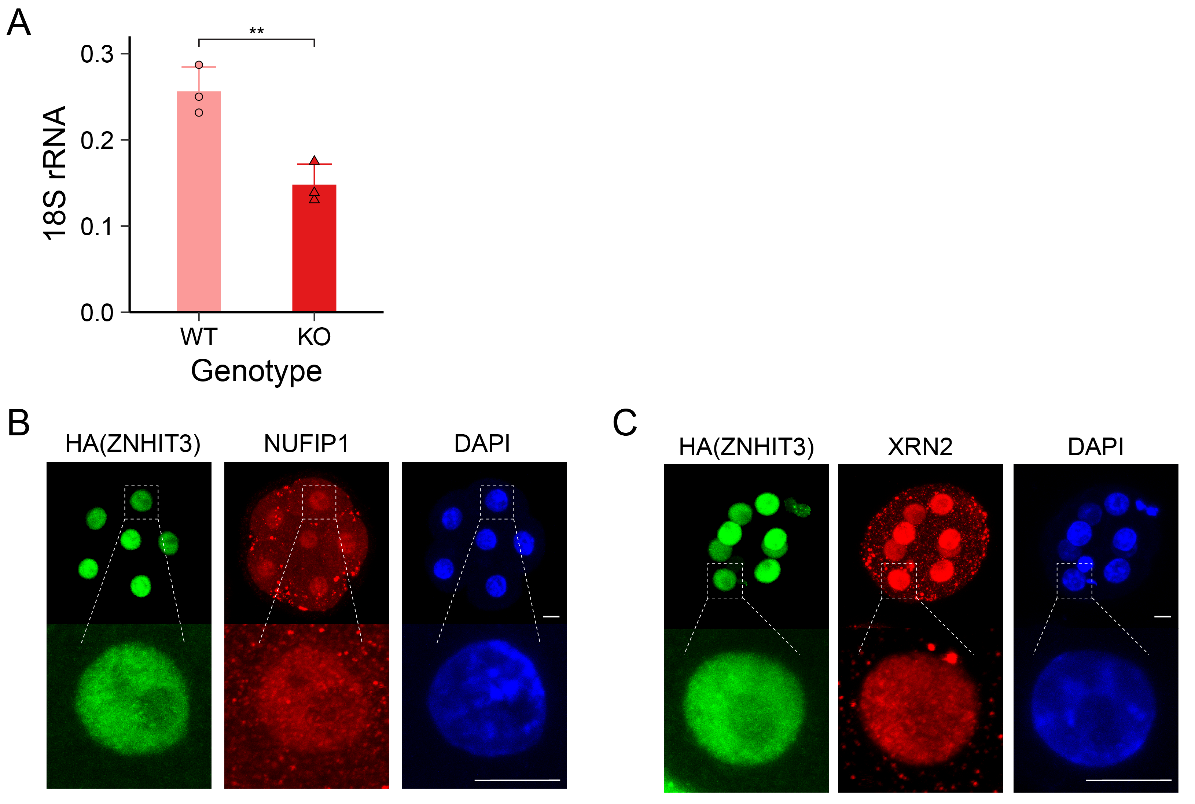


**Figure S5.** *Znhit3* ablation leads to reduction of 18S ribosomal RNA and ZNHIT3 has similar cellular localization with NUFIP1 and XRN2. A) qRT-PCR examination of 18S rRNA level in control and *Znhit3^-/-^* morulae. ERCC is supplied to extracted RNA from embryos and used as external control. Note qRT-PCR results correlate well with that from single embryo RNA-seq. B,C) Immunofluorescence showing cellular localization of ZNHIT3, NUFIP1 and XRN2. *Znhit3^KI/-^* female mice were used to collect embryos after intercrossing. ZNHIT3 has similar cellular localization with NUFIP1, another key component of box C/D snoRNP complex and XRN2, a RNase that is responsible for mRNA splicing. These results corelates well with the IP/MS experiment (Table S3) indicating ZNHIT3 is another component of box C/D snoRNP complex and may recruit pre-mRNA splicing complex.

**
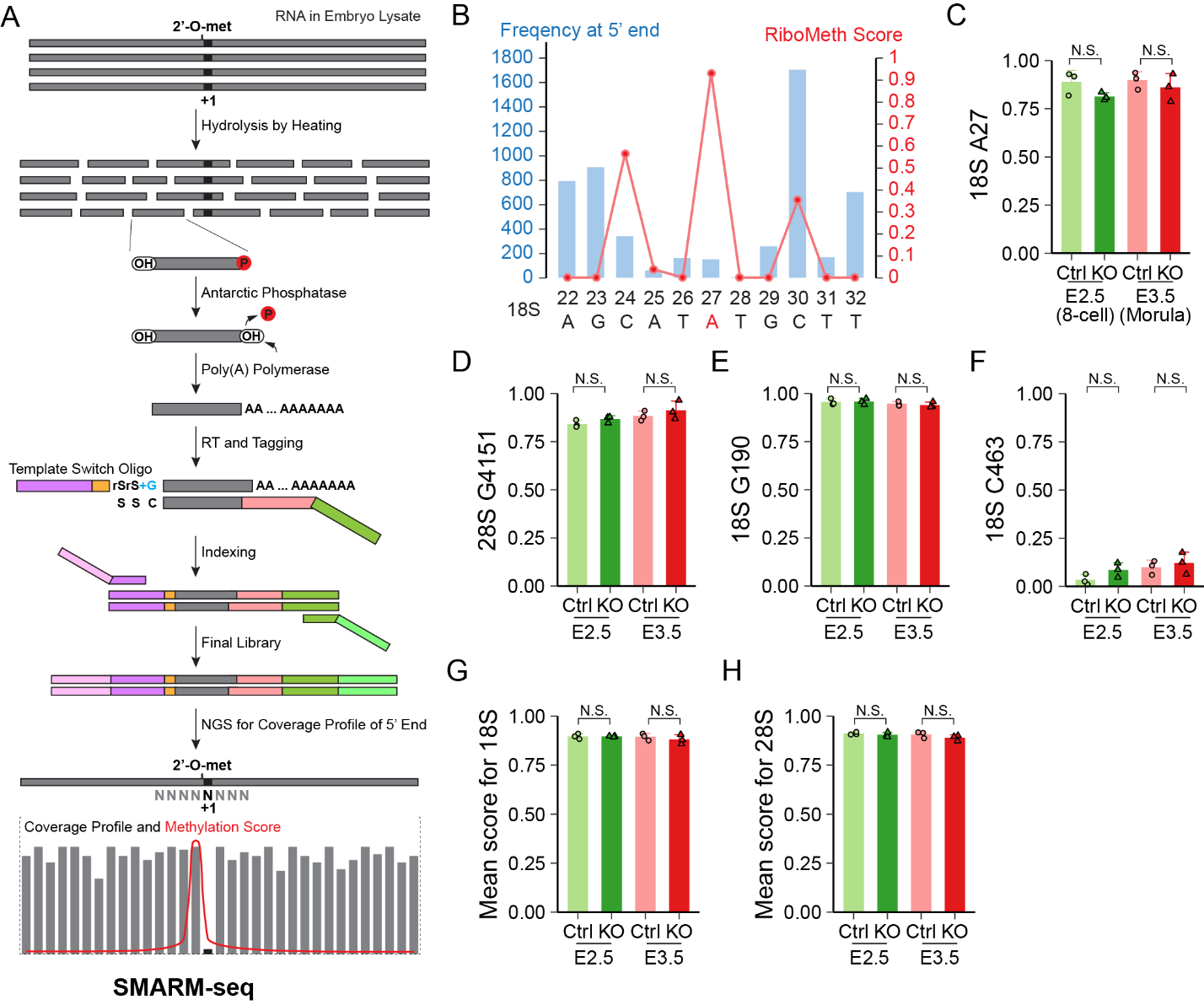
Figure S6.** *Znhit3* ablation leads to marginally reduced 2’-O-methylation of ribosomal RNA. A) Scheme of the SMARM-seq method that is applied to detect 2’-O-methylation using tens of embryos. B) Frequencies (left) of several 18S bases at the 5’ end of SMARM-seq reads. The corresponding RiboMeth score for each position is also shown on the right. One base (position) with RiboMeth Score > 0.8 is considered 2’-O-methylated (*e.g.*, base ‘A’ at position 27 in this panel). C) RiboMeth score of 18S rRNA A27 base in *Znhit3^-/-^* or control embryos with *Znhit3^FLAG-HA^* allele at the indicated developmental stages. D-F) Same as in C, but for 3 other positions, 28S G4151, 18S G190 and 18S C463. Surprisingly, 2’-O-methylation of 18S C463 was reported to be conserved among many species but is hardly detected by SMARM-seq. G,H) Mean scores of 2’-O-methylation for all bases of 18S and 28S rRNA as detected by SMARM-seq in mouse embryos at different developmental stages. There are no significant differences between *Znhit3^-/-^* and control samples.


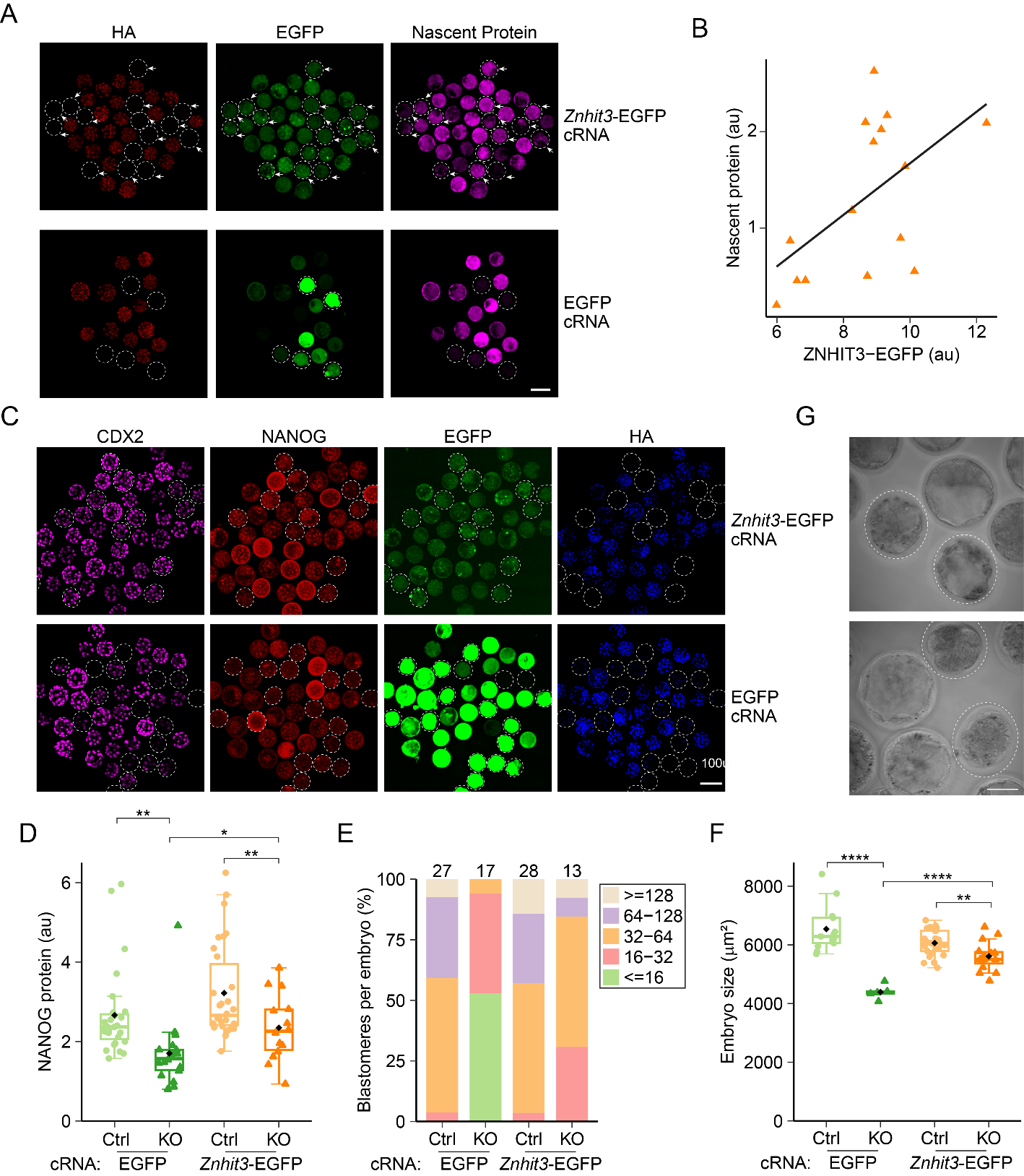


**Figure S7.** *Znhit3* cRNA rescues defects in *Znhit3^-/-^* embryos. A) Images of nascent proteins in *Znhit3^-/-^* and control embryos at E4.5 after zygotic injection of EGFP or *Znhit3*-EGFP cRNA. Scale bar, 100 μm. B) Relationship between expression of *Znhit3*-EGFP cRNA and nascent protein levels in *Znhit3^-/-^* embryos. Note higher expression of *Znhit3* cRNA leads to better recovery of protein synthesis in the embryos. C) Images of CDX2, NANOG proteins in *Znhit3^-/-^* and control embryos at E4.5 after zygotic injection of EGFP or *Znhit3*-EGFP cRNA. CDX2 protein levels are quantified in Figure 7F and NANOG protein levels are quantified in D. E) Number of blastomeres in *Znhit3^-/-^* and control embryos at E4.5 after zygotic injection of EGFP or *Znhit3*-EGFP cRNA. F) Size of *Znhit3^-/-^* and control embryos at E4.5 after zygotic injection of EGFP or *Znhit3*-EGFP cRNA. Embryo size reflects the area taken after vertical projection of all blastomeres within one embryo without counting the zona pellucida region. G) Enlarged DIC images in Figure 7E. Scale bar, 100 μm.

**Tables S1 to S5 are included as separate Excel (.xlsx) files.**

**Table S1.** seRNA-seq gene count matrix from 4-cell to early blastocyst stage.

**Table S2.** scRNA-seq gene count matrix of blastomeres from 8-cell embryos.

**Table S3.** IP/MS results of possible ZNHIT3 binding proteins.

**Table S4.** RiboMeth scores of rRNA bases as determined by SMARM-seq.

**Table S5.** Primers and Oligos.

**Data S1 is included as a separate .zip file.**

**Data S1**. Images of all Z-slices in immunofluorescence experiments related to Figure 5A,D; Figure 6A,C; Figure 7E; Figure S7C.
